# Osteopontin stabilization and collagen containment slows amorphous calcium phosphate transformation during human aortic valve leaflet calcification

**DOI:** 10.1038/s41598-024-62962-8

**Published:** 2024-05-28

**Authors:** Mayandi Sivaguru, Shumpei Mori, Kyle W. Fouke, Olujimi A. Ajijola, Kalyanam Shivkumar, Ashok Z. Samuel, Rohit Bhargava, Bruce W. Fouke

**Affiliations:** 1 Cytometry and Microscopy to Omics Facility, Roy J. Carver Biotechnology Center, University of Illinois at Urbana-Champaign, Urbana, IL USA; 2 Earth Science & Environmental Change, School of Earth, Society and the Environment, University of Illinois at Urbana-Champaign, Urbana, IL USA; 3 Cardiac Arrhythmia Center and Neurocardiology Research Program of Excellence, David Geffen School of Medicine, UCLA Health, University of California Los Angeles, Los Angeles, CA USA; 4 Department of Earth and Planetary Sciences, Jackson School of Geosciences, University of Texas at Austin, Austin, TX USA; 5 Department of Bioengineering, Grainger College of Engineering, University of Illinois at Urbana-Champaign, Urbana, IL USA; 6 Beckman Institute for Advanced Science and Technology, University of Illinois at Urbana-Champaign, Urbana, IL USA; 7 Department of Chemical and Biological Engineering, Grainger College of Engineering, University of Illinois at Urbana-Champaign, Urbana, IL USA; 8 Cancer Center at Illinois, University of Illinois at Urbana-Champaign, Urbana, IL USA; 9 Biomedical and Translational Sciences, Carle Illinois College of Medicine, University of Illinois at Urbana-Champaign, Urbana, IL USA; 10 Department of Evolution, Ecology and Behavior, School of Integrative Biology, University of Illinois at Urbana-Champaign, Urbana, IL USA; 11 Roy J. Carver Biotechnology Center, University of Illinois at Urbana-Champaign, Urbana, IL USA

**Keywords:** Amorphous calcium phosphate (ACP), Hydroxyapatite (HAP), Aortic valve, Cardiovascular calcification, Coalescing spherules, Nodules, Collagen alteration, Collagen containment, Cholesterol, GeoBioMed, Lipids, Osteopontin, Spherules, Super-resolution autofluorescence, Calcification, Valvular disease

## Abstract

Calcification of aortic valve leaflets is a growing mortality threat for the 18 million human lives claimed globally each year by heart disease. Extensive research has focused on the cellular and molecular pathophysiology associated with calcification, yet the detailed composition, structure, distribution and etiological history of mineral deposition remains unknown. Here transdisciplinary geology, biology and medicine (GeoBioMed) approaches prove that leaflet calcification is driven by amorphous calcium phosphate (ACP), ACP at the threshold of transformation toward hydroxyapatite (HAP) and cholesterol biomineralization. A paragenetic sequence of events is observed that includes: (1) original formation of unaltered leaflet tissues: (2) individual and coalescing 100’s nm- to 1 μm-scale ACP spherules and cholesterol crystals biomineralizing collagen fibers and smooth muscle cell myofilaments; (3) osteopontin coatings that stabilize ACP and collagen containment of nodules preventing exposure to the solution chemistry and water content of pumping blood, which combine to slow transformation to HAP; (4) mm-scale nodule growth via ACP spherule coalescence, diagenetic incorporation of altered collagen and aggregation with other ACP nodules; and (5) leaflet diastole and systole flexure causing nodules to twist, fold their encasing collagen fibers and increase stiffness. These in vivo mechanisms combine to slow leaflet calcification and establish previously unexplored hypotheses for testing novel drug therapies and clinical interventions as viable alternatives to current reliance on surgical/percutaneous valve implants.

## Introduction

The evolutionary success of invertebrate and vertebrate organisms through geological time has relied on their ability to harness the precipitation of thermodynamically unstable amorphous calcium phosphate (ACP) before it spontaneously transforms into crystalline hydroxyapatite (HAP)^1,2^. While ACP calcification is fundamental to an organism’s ability to precipitate essential hard parts such as bone and teeth, the capacity of ACP to morphologically shape-shift and atomically rearrange also results in various soft tissue pathologies^3^. This affinity for compositional flexibility reflects the dynamic nature of transient calcium phosphate compounds that lead to ACP biomineralization and eventual transformation toward HAP, a process in which nanometer-scale particles with short-range ionic order^4,5^ aggregate and undergo repeated events of precipitation, dissolution and reprecipitation (*diagenetic phase transitions*^6^). The complex physical, chemical and biological interactions controlling ACP (Ca_x_(PO_4_)_z_·nH_2_O, n = 3–4.5; 15–20 wt% H_2_O)^3,4^ calcification are strongly influenced by solution chemistry (pH, saturation state and calcium/phosphate [Ca/P] concentrations), availability of H_2_O^4^ and activity of extracellular matrix proteins and peptides that stabilize ACP and prevent its transformation into crystalline HAP^7–10^.

There is mounting unmet need to discover new clinical therapies for the prevention and treatment of calcification in the human circulatory system^11–16^. This process of cardiovascular calcification is a significant factor in the more than 18 million lives claimed globally each year by heart disease^12^. Stenosis of vasculature associated with blood flow restriction and heart valve calcification that leads to cardiac dysfunction has long afflicted humankind. Hardening of the arteries (atherosclerosis) has been observed in 4000 year-old human mummies from ancient cultures around the world^17^. Leonardo Da Vinci in 1513-14 AD confirmed the narrowing of arteries as “the thickening of coats of these veins” in his studies of the human heart^18^. In modern society, cardiovascular calcification continues to be a common health disorder in people of all ages, genders and ethnic backgrounds, is associated with other comorbidities^19,20^, and is the most prevalent form of heart disease in patients 65 and older^12,21^. Yet beyond invasive valve implants, there are no viable alternative drug therapies or clinical treatment options available^12,22^.

Previous research on aortic valve calcification (also called calcific aortic valve disease; CAVD^23^) has primarily focused on cellular and molecular pathophysiology processes, including extracellular matrix biochemistry and biomechanics, but has not specifically targeted the etiological processes recorded by the calcification deposits themselves^7,11–15,23,24^. This is because standard microscopy techniques for pathological screening include stains that dissolve ACP and/or transform ACP to HAP in tissue sections, while x-ray diffraction cannot resolve the short-range ordering of ACP. Since 1975, several comprehensive reviews^12,13,15,25^ refer to four basic research studies^26–29^ that have identified ACP as the primary agent of aortic valve and arterial calcification by combining electron diffraction, standard microscopy and microprobe analyses with optical microscopy on unstained histological cryosections. Now, a half century later, rigorous examination specifically targeting the role of ACP in cardiovascular calcification remains to be completed^12,14,25,29^. This is in sharp contrast to long-running recognition of the fundamental importance of ACP biomineralization in bone, teeth and kidney stones^4,30–32^, as well as a wide variety of other bioengineering applications that have used multiple nano- and micro-scale analytical techniques to characterize ACP^3,32–34^.

### GeoBioMed approach

The present study specifically targets calcification deposits formed in aortic valve leaflet tissues utilizing a transdisciplinary approach called *GeoBioMed* that combines concepts and techniques from the fields of geology, biology, and medicine^6^. These analyses of original unaltered and calcified (*diagenetically altered*^6^) aortic valve leaflet tissues reveal the distribution, composition and developmental record (paragenetic sequence^6^) of ACP, ACP at the initial stages of transformation toward hydroxyapatite (HAP), and cholesterol biomineralization within the context of tissue structure, cellular function, biomolecular activity and patient medical history. Collectively, this GeoBioMed evidence establishes a detailed history of soft tissue pathological calcification events, each stage of which occurs sequentially and/or simultaneously and to differing extents of reaction^35^ throughout each aortic valve leaflet. The paragenetic sequence spans from initial unaltered tissues through advanced calcified stages of collagen fiber and smooth muscle cell (SMC) myofilament calcification, stabilization by osteopontin (OPN), nodule formation and containment mechanisms and rotation during flexure that results in stiffening. The result is a previously unexplored roadmap for future development of untested GeoBioMed clinical therapies for the prevention and treatment of aortic valve leaflet calcification.

**Figure 1:**
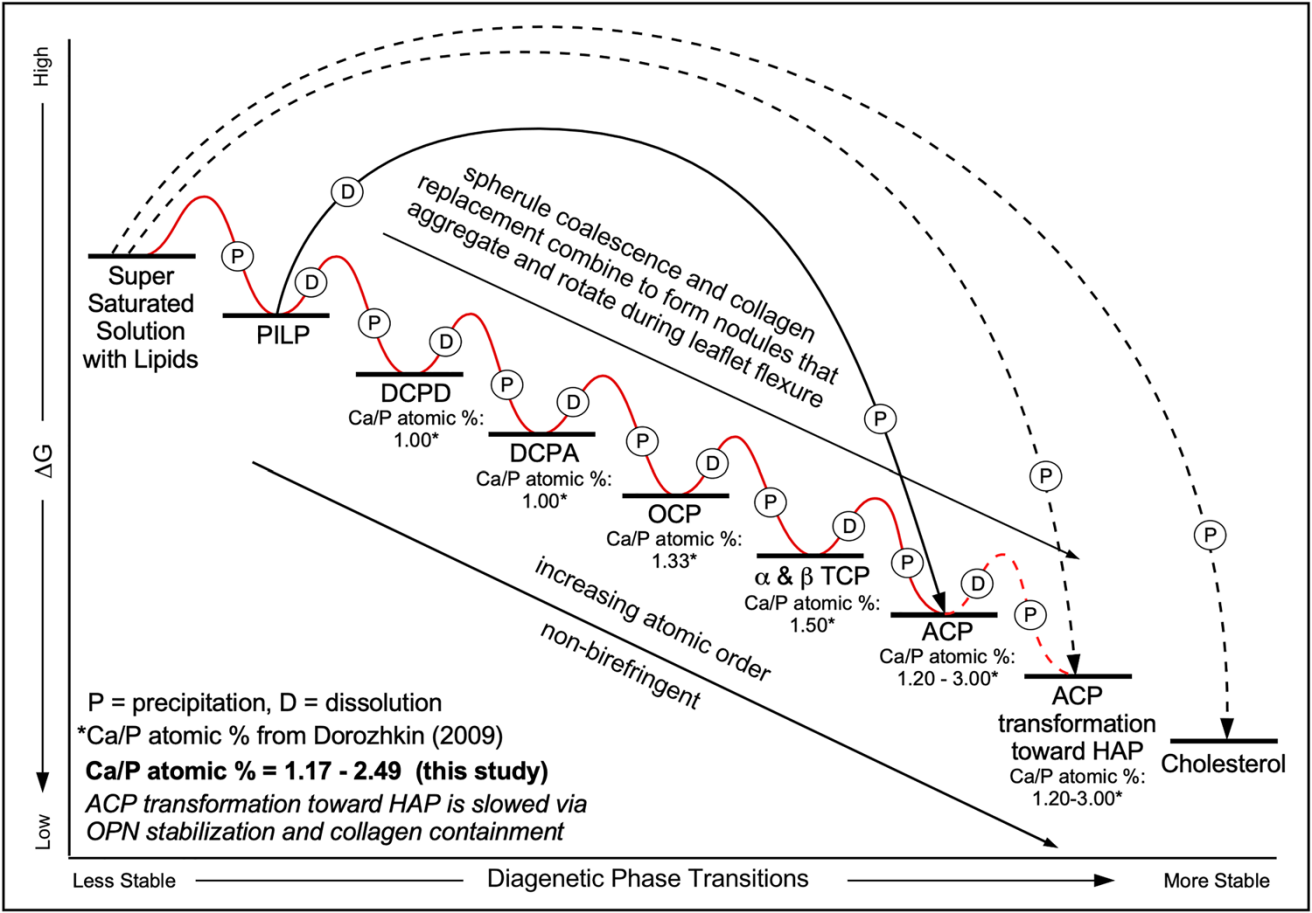
Diagenetic phase transitions during calcification of human aortic valve leaflets. Diagenetic phase transitions and associated changes in Gibbs free energy (ΔG) during simultaneous ACP, ACP transformation to HAP and cholesterol diagenetic phase transitions. Calcium/phosphate (Ca/P) atomic ratios are listed for each the intermediate metastable (transient) ACP phases^4,37–41^. Graph illustrates: (1) unconventional (kinetic) step wise ACP diagenetic phase transitions (solid black and red lines)^38^; (2) initial stages of ACP transformation toward HAP (dashed red line); (3) direct (thermodynamic) precipitation of HAP from saturated solution with lipids (dashed black line); and (4) direct precipitation of cholesterol from saturated solution with lipids (dashed black line). OPN stabilization and collagen containment combine to slow the transformation of ACP to HAP. ACP = amorphous calcium phosphate. HAP = hydroxyapatite. CPOL = circular polarization. PILP = polymer-induced liquid-precursor. DCHD = dicalcium phosphate dihydrate. DCPA = dicalcium phosphate anhydrous. OCP = octacalcium phosphate. α and β TCP = tricalcium phosphate. HAP = hydroxyapatite.

For the purposes of the present study, the term ACP is used to collectively refer to: (1) the transient amorphous phases of calcium phosphate that include dicalcium phosphate dihydrate (DCHD), dicalcium phosphate anhydrous (DCPA), octacalcium phosphate (OCP), α and β tricalcium phosphate (TCP) and ACP itself^3,4^; and (2) ACP deposits that, while still amorphous and non-birefringent, exhibit low order transformations toward HAP that begin to exhibit geometric (euhedral) forms^36^ (Fig. 1). Multiple previous medical and engineering studies^3,4,26–30,32–34,36^ have established an extensive analytical data base with which to consistently and accurately identify ACP and cholesterol biomineralization deposits in histological cryosections and petrographic epoxy impregnated sections. Optical, electron, laser and x-ray microscopy and spectroscopy evidence collected in the present study utilize this literature base as a comparative standard to confirm that the biomineralization deposits formed in the aortic valve leaflet tissues are predominantly composed of ACP and a minor component of crystalline cholesterol (Fig. 1). Internal controls for characterizing calcified tissues are provided by analyzing pristine non-calcified portions of the aortic valve leaflet tissues that are common throughout each histological cryosection and petrographic section.

**Figure 2:**
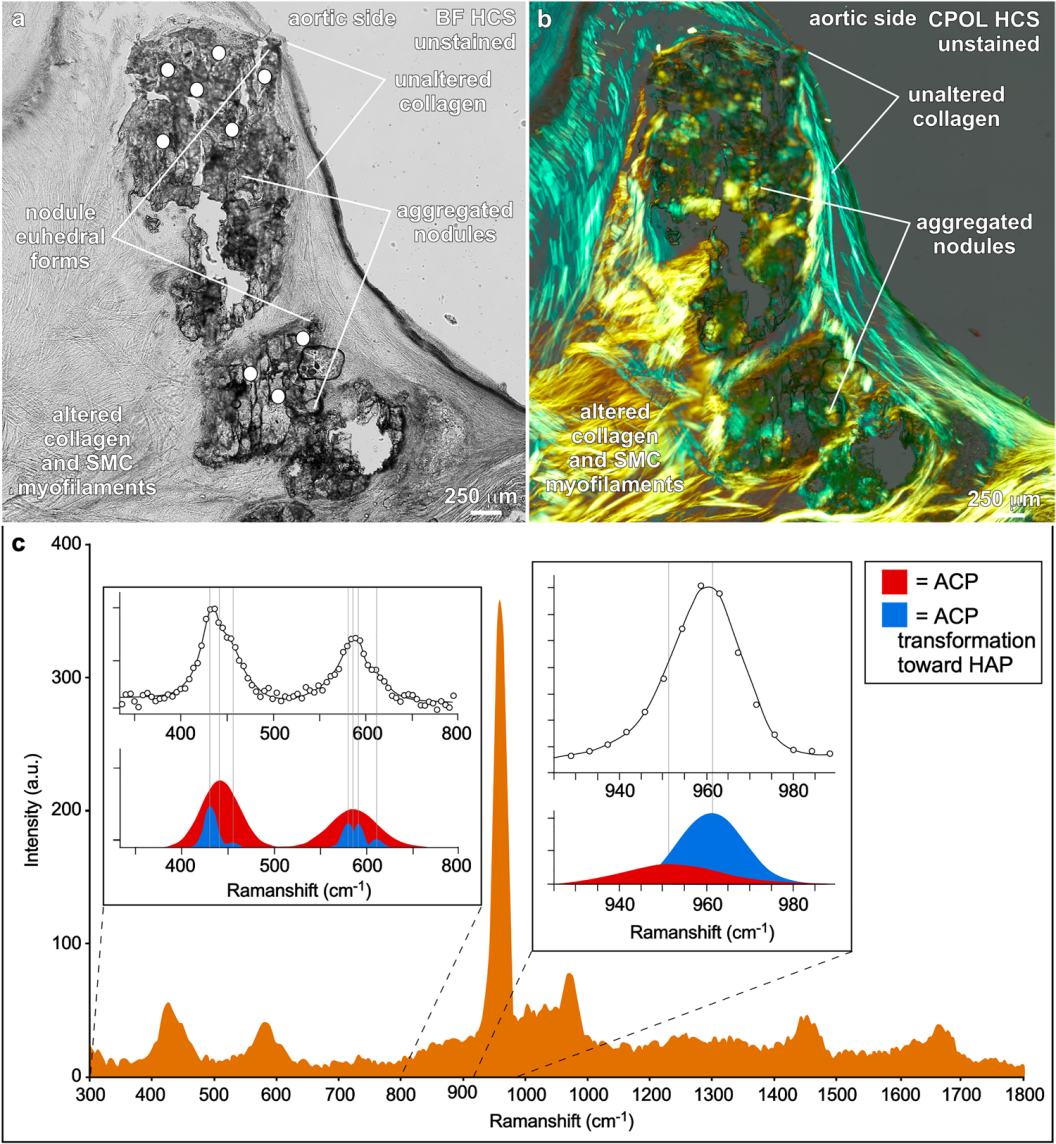
Raman spectroscopy of nodules within human aortic valve leaflet tissues in histology cryosections embedded in ultrapure water. (**a**) and **(b**)**,** Paired high resolution BF (**a**) and CPOL (**b**) images showing the location (white dots) of Raman spot analyses (n = 9) of nodules that formed within original and altered collagen fibers and SMC myofilaments comprising the fibrosa tissue layer at the aortic margin of calcified aortic valve leaflets. Some nodules are beginning to exhibit geometric (euhedral) forms. Additional low magnification contextual images presented in SI Fig. 10. (**c**), A representative 300–1800 cm^-1^ Raman baseline spectrum from the nine spot analyses shown in (**a**). Expanded spectral regions are shown at 400–700 and 925–985 cm^-1^. Broad red and blue filled peak areas correspond to ACP and ACP transformation toward HAP Raman bands, respectively, resolved by performing peak fitting analyses [see experimental Sect. ^42^ for details]. Unfilled circles and solid black line represent raw data and their fit. These Raman analyses, in combination with the suite of other integrated analyses conducted in the present study, indicate that the nodules are composed of metastable ACP and ACP at the threshold of transforming toward HAP. ACP = amorphous calcium phosphate. HAP = hydroxyapatite. SMC = smooth muscle cell.BF = bright field. HCS = histology cryosection. CPOL = circular polarization.

Several lines of evidence collected in the present study indicate that the biominerals responsible for aortic valve calcification and resulting leaflet stenosis are composed of multiple transient forms of ACP, ACP at the threshold of transformation to HAP and cholesterol. These analyses include (Table 1; Figs. 1, 2): (1) energy dispersive elemental analyses (EDAX)^4^ indicating calcium (Ca) to phosphate (P) atomic % compositions (Ca/P) of 1.17 to 2.49; (2) Raman spectroscopy^42,43^ exhibiting broad peak overlap ranges of 400–500, 550–700 and 925–985 cm^−1^; and (3) absent (extinct) to extremely low birefringence under high resolution circular polarization (CPOL)^6,36^, while simultaneously exhibiting geometric (euhedral) forms under bright-field (BF) and CPOL microscopy. The aortic valve leaflet biomineralization deposits also exhibit characteristic mottled and generally poorly defined diffuse and shapeless textures of ACP. This is confirmed with the integration of micro-computed tomography (Micro-CT) imaging, high resolution BF, CPOL^6^ and transmitted light photomultiplier tube (TPMT)^6^ microscopy, and environmental scanning electron microscopy (ESEM)^6^ (Table 1). In addition, organic molecules coating biomineral individual layers are entrapped by each ensuing layer of ACP deposition, creating a high fidelity nm-scale microstratigraphy record of calcification^6^. These processes are documented with confocal autofluorescence (CAF)^6^, widefield fluorescence (WF)^6^, super resolution auto fluorescence (SRAF)^6^, and super resolution induced fluorescence (SRIF) microscopy^6^ using the Alexa 647 antibody against osteopontin (OPN)^8,10^. While OPN is a catalyst of HAP precipitation in non-phosphorylated form, it has also been shown that phosphorylated OPN acts as a strong inhibitor of ACP deposition^8–10,44,45^. Furthermore, Oil Red O (ORO) stain under BF is used to identify the presence of lipids and Alizarin Red S (ALZ) stain under BF to identify calcium in ACP. In addition, the well-defined acicular to lathe-shape crystalline structure of cholesterol under BF exhibits strong birefringence under CPOL^46^, which is further substantiated with the comprehensive suite of analyses presented in Table 1.

**Table 1:** Integrated light, laser, electron and x-ray analyses used to identify and spatially map the distribution of calcification within human aortic valve leaflets.

System components	Analyses (resolution)											
	Histology cryosection	Petrographic section	CT (600 μm) micro-CT (3 μm)	ESEM (5nm)	EDAX (1 μm) ca/p atomic % + / − SD n = 9	Raman (1 μm) spectra cm^−1^	BF (240 nm) TPMT (240 nm)	BF (240 nm) ORO & ALZ	CPOL (240 nm)	CPOL (240 nm) ALZ	SRAF (140 nm) DAPI	SRAF (140 nm) OPN
Aortic value leaflet tissuse	**✓**	**✓**	**✓**	**✓**	**✓**	**✓**	**✓**	**✓**	**✓**	**✓**	**✓**	**✓**
Lipids	✓	na	na	na	Na	na	na	na	na	na	Red	na
Nuclei	✓	na	na	na	Na	na	na	na	na	na	Blue	na
Osteopontin OPN	✓	na	na	na	Na	na	na	na	na	na	na	Green
Cholesterol crystals	✓	✓	✓	✓	✓	na	✓	Red	1st–2nd order*	1st–2nd order*	Black	✓
Original collagen	✓	✓	✓	✓	Bd	na	✓	na	2nd order*	2nd order*	Blue–green	✓
ACP replaced collagen	✓	✓	✓	✓	1.17–2.49 + / − 0.38	943–958	✓	Red	2nd order*	2nd order*	Red–yellow	✓
ACP nodule	✓	✓	✓	✓	1.47–1.91 + / − 0.15	943–958	✓	Red	2nd order*	2nd order*	Gray–green	✓

Summary of analyses of each of the primary calcification components and analyses. Summary of analyses used to characterize the primary components of aortic valve leaflet calcification.

*ACP* amorphous calcium phosphate, *OPN* osteopontin, *CT* computed tomography, *Micro-CT* micro-computed tomography, *ESEM* environmental scanning electron microscopy, *EDAX* energy dispersive spectroscopy, *Raman* Raman spectroscopy, *BF* brightfield microscopy, *TPMT* transmission photomultiplier tube, *ORO* Oil Red O red stain for lipids, *ALZ* Alizarin Red S stain for calcium, *DAPI* 4′,6-diamidino-2-phenylindole stain for DNA, *OPN* Alexa 647 antibody green stain for osteopontin, *CPOL* circular polarization, *CPOL ALZ* birefringence induced by ALZ stain for calcium, *SRAF* super resolution autofluorescence, *SRIF* super resolution induced fluorescence.

✓ bold = analysis completed, ✓ unbold = delected, na = not applicable, bd = below detected, * = Zesis 2015 Michel-Levy interference colour chart.

## Materials and methods

### Human aortic valve leaflet samples

All methods in this study were carried out in accordance with guidelines and regulations in a basic medical research study plan that was reviewed and approved by the UCLA Health Institutional Review Board (IRB 19-000624). Three human cadaveric (deceased patient) hearts were analyzed from a cohort of 26 hearts reposited at the Cardiac Arrhythmia Center and Neurocardiology Research Program of Excellence at UCLA Health. Written informed consent was obtained from all UCLA health patient participants as part of the OneLegacy Foundation and the NIH SPARC Program, which formed the basis for obtaining cadaveric donor hearts for research and for funding this effort. These cadaveric hearts were chosen based on their differential extent of calcification as described below. After 24 h pressure perfusion and fixation with 4% paraformaldehyde, samples were stored in 1 × PBS and 0.02% Sodium Azide at 4 °C until analysis. The heart donor population was 54 ± 10 years old, 65% male, and exhibited 31 ± 12% aortic calcification, 15 ± 4% aortic valve calcification, and 62 ± 7% coronary arterial calcification as calculated from CT scans.

### Leaflet dissections and preparation of histological cryosections and petrographic sections

Legacy hearts chosen for analysis were first thoroughly documented with high-resolution photography using a Nikon D850 camera at UCLA Health. The three-dimensional (3D) distribution of calcification within each whole legacy heart prior to dissection was determined with: (1) 3D CT scans at a resolution of 600 μm on an CT (SOMATOM Definition AS, Siemens Healthcare, Forchheim, Germany) at UCLA Health; and (2) 3D micro-computed tomography (Micro-CT) scans at a resolution of 3 μm on a North Star X 5000 at the Roy J. Carver Biotechnology Center (CBC) and Carl R. Woese Institute for Genomic Biology (IGB) at the University of Illinois Urbana-Champaign (Illinois). CT and Micro-CT 3D x-ray scan data sets were then rendered and evaluated on a Hewlett Packard-Z-10 Workstation. These coupled CT and Micro-CT scans were used to guide dissections of each of the three aortic valve leaflets (left coronary, right coronary and non-coronary) and precisely record their 3D spatial orientation and positioning within the aortic valve. Dissections were completed at UCLA Health (which included additional high-resolution photography) with minor follow-up dissections in the Illinois CMtO CBC laboratory. All dissected samples were fixed in 10% formalin, gently washed in deionized water and transferred to 50 mL falcon tubes containing 1 × PBS and 0.1% Sodium Azide and stored at 4 °C until analysis. Prior to sectioning, dissected leaflets were scanned again on a North Star X-5000 Micro-CT at the CBC and IGB, as well as on a Rigaku HX130 Micro-CT at the Illinois Beckman Institute for Advanced Science and Technology. Individual leaflets were then further dissected, frozen in liquid nitrogen-cooled Isopentane, embedded in optimum cutting temperature (OCT; Tissue Tek, Sakura Fine Tek, USA) cryopreservation medium (non-coronary leaflets) and kept at − 80 °C until sectioning. From which a total of 30 serial histology crysections for tissue analysis were made at the Histology Laboratory at the Illinois College of Veterinary Medicine. Histology sections were glass mounted on an Epredia NX 70 cryostat at − 20 °C, after which the sections were stored at − 80 °C until analysis (uncovered, 20–30 μm-thick for three-dimensional [3D] analysis). An intact aortic valve leaflet (right) was air dried at 37 °C for 24 to 48 h in an Epredia warm air oven. From which, two petrographic glass-mounted thin Sects. (25 μm-thick, doubly polished) for mineralogical analyses were prepared at Wagner Petrographic Ltd. (Lindon, UT) using low viscosity cathodoluminescence-resistant epoxy and precise cutting angles to contextually capture the progression of nodule calcification within the leaflet tissue.

### Immunofluorescence labeling of osteopontin (OPN) protein

The sequentially made histological cryosections were segregated into cohorts, which included: (1) unstained sections; (2) sections stained with Alizarin Red (ALZ) for calcium in ACP, Oil Red O (ORO) for lipids (both are contrasting stains for bright field microscopy) and a fluorescence 4′,6-diamidino-2-phenylindole stain (DAPI) for DNA (for fluorescence, confocal and super resolution microscopy); and (3) sections for immunohistochemical fluorescence labeling for the osteopontin (OPN) protein (fluorescence, confocal and super resolution microscopy). For immunohistochemical labeling, sections were removed from storage at -80 °C and placed on a slide holder in a C1000 Touch Bio-Rad thermal cycler set at 37 °C for one minute, then incubated with prechilled HPLC grade methanol for 30 min at − 20 °C. Sections were then immediately removed and hydrated in phosphate buffered saline containing 2% Triton-X-100 (PBST) for 30 min. Sections were blocked with IT Signal FX (I36933-ThermoFisher, Carlsbad, CA) for 30 min in a dark room to remove any unspecific binding of antibodies followed by incubation with an OPN antibody (CoraLite Plus 647 conjugated Osteopontin Rabbit polyclonal antibody (CL647-22952, Proteintech, Rosemont, IL) at 1:100 dilution with PBST and IT Signal FX (10%) for overnight incubation in a humid chamber. Sections were then washed three times in PBST, mounted in Prolong Gold (P36935-ThermoFisher, Carlsbad, CA), an antifade mounting medium containing DAPI. After wicking away excess mounting medium, sections were sealed with a cover glass and kept in the dark overnight for curing until reaching a higher refractive index (~ 1.4) to enable index matching high-resolution imaging with oil immersion objectives. These covered sections were then sealed with quick dry nail polish and stored at 4 °C, until analysis.

### High-resolution imaging of aortic valve leaflets using multiple modalities

Petrographic epoxy impregnated sections were imaged unstained. However, the other consecutive histological cryosections of aortic valve leaflets were labeled with multiple visible contrast stains (for bright field microscopy) such as Alizarin Red S (ALZ) for Ca in ACP, Hematoxylin and Eosin stains for cellular morphology and Oil Red O (ORO) for lipids. The intermittent serial sections, not labeled with visible stains, were mounted with Prolong Gold antifade reagent containing DAPI (ThermoFisher Scientific, Carlsbad, CA), which labels nuclei/double stranded DNA in the sectioned samples and cured for 24–48 h to reach a refractive index of ~ 1.4 and mounted with a 170 μm-thick cover glass to improve the index matching, signal integrity and image quality, when especially using oil immersion objectives as described above.

The present study utilizes multiple optical modalities (Table 1) as described previously^26^, which include BF, TPMT, CPOL, CAF, WF, SRAF and SRIF modalities that were completed on a custom built Carl Zeiss LSM 980 Spectral NLO Airyscan II Super resolution system (Carl Zeiss, Oberkochen, Germany) housed in the CBC. This microscope system is mounted with a Carl Zeiss Axiocam 712 color camera for brightfield and polarization optical modalities and a Hamamatsu Orca-Flash4.0 digital CMOS camera for fluorescence images excited by Excelitas, Xylis broad spectrum LED illumination light source. This suite of optical hardware uniquely enabled multimodal images (BF, POL, CPOL, TPMT, WF, CAF, SRAF and SRIF), at precisely the same locations of interest under multiple magnification. Large areas of samples are also tiled (using a factory-built Zeiss automated XY stage) to capture contextual visualization of the whole section, as well as 3D images using Z-stack functionalities across a broad range of magnifications (10 × Plan Neofluar:0.45 NA; 20 × Plan Apochromat: 0.75 NA and 0.8 NA; 40 × Plan Apochromat Oil immersion: 1.4 NA; 100 × Plan Apochromat Oil immersion: 1.49 NA). Compared to conventional crossed Nicol polarization, the current utilization of CPOL quarter-wave plate (λ/4 wide spectrum compensator covering 0–30 λ together with a 570 nm retardation plate), which enhanced the birefringence of all crystals without depending on their extinction axis and added another order of birefringence color spectrum as per Zeiss Michel-Levy’s interference chart (2015; Supplementary Fig. 7). Under the CAF and SRAF/SRIF modalities, we used 405 nm (emission collected between 410–460), 488 nm (emission collected between 500–550) and 561 nm excitations (emission collected between 570–615) to collect the entire visible emission spectrum of autofluorescence signatures from both organic and inorganic crystalline architecture. Since the tissue is already autofluorescent, the OPN antibody conjugated dye was selected specifically to be distinct at 647 nm excitation to avoid any overlap with the autofluorescence (excitation 647 nm and emission collected between 650–720 nm). In some instances, a few SRAF and SRIF channels were merged after appropriate pseudo-coloring of the individual channels. While CAF provided a diffraction limited resolution of ~ 250 nm, the SRAF and SRIF images yielded 140 nm super resolution images. The pixel resolutions of the images were between 20 to 300 nm for most images under these two modalities. The TPMT images are obtained using the shortest wavelength available for retrieving highest optical resolution (405 nm laser) and the corresponding resolutions were ~ 180–200 nm under the 100 × 1.49 NA oil immersion objective. The tiles and Z-stack were optimized with multiple supporting focal points, stitched using the Carl Zeiss Zen (Carl Zeiss, Oberkochen, Germany) stitching module and the Z-stack slices were around 130 nm per slice and a range of 5–15-micron depth images were 3D projected using the 3D Surpass algorithm in the 3D visualization and rendering program Imaris (version 10.0, Oxford Instruments, Carteret, NJ).

### Environmental scanning electron microscopy (ESEM) and energy dispersive elemental analysis (EDAX)

ESEM of both intact human aortic valve leaflets containing nodules and consecutive sections from the same blocks of tissue. Leaflet samples used for standard histological cryosectioning were critical point dried using hexamethyldisilazane (HMDS) followed by sputter coating with gold palladium (Au/Pd) target (Denton DESK II TSC, Moorstown, NJ). Samples were then imaged under vacuum in a FEI Quanta FEG 450 FESEM (Hillsboro, OR), housed in the Illinois Beckman Institute for Advanced Science and Technology, under multiple magnifications on cryosectioned samples as well as undisturbed intact aortic valve leaflets after mounting a chuck with carbon tape. As described previously^6,47^, ESEM imaging was done under the default setting of an ~ 10 mm working distance, 20 kV beam with a spot size of 4.0 nm, and multiple magnifications with a dwell time of 300 ns. On histological cryosections and petrographic sections, elemental maps and energy dispersive elemental analyses (EDAX; n = 9) with a spot size of 3 μm at each sample location (Supplementary Data Fig. 2) to specifically analyze Ca, P and Mg, the elements that are indicative of ACP calcification. Spot analysis locations included unaltered collagen, and individual ACP spherules and crusts on diagenetically unaltered collagen. The EDAX Ca/P atomic ratios were calculated and graphed in Microsoft Excel.

### Raman spectroscopy of calcified leaflets

Calcification mineralogy was assessed with a WiTec Alpha300 RSA Raman imaging microscope system (WiTec, Nashville, TN) housed in the IGB with a 532 nm-wavelength laser. A 100 × air objective (LU Plan Fluor 0.8 NA) capable of providing submicron spatial resolution was used to record Raman spectra from 5 μm-thick histology cryosections mounted in ultrapure water of the aortic valve leaflets. Spectra were collected with a 1–10 mW laser power and a 1 s exposure time. An optical fiber, providing an effective pinhole of 50 μm, was used to transmit the optical signal to a WiTec UHTS400 spectrometer and WiTec Peltier cooled CCD camera. Peak fitting analysis was performed by assuming a Gaussian line profile (Type: Gauss (position cm*-1*, FWHM ∆ cm*-1*); position 431, FWHM = 16.2; position 442, FWHM = 48.0; position 454, FWHM = 14.4; position 581, FWHM = 14.3; position 586, FWHM = 60.0 cm^−1^; position 592, FWHM = 11.8; position 611, FWHM = 14.2; position 952, FWHM = 25.0; position 961, FWHM = 16.7). This analysis reveals all expected phosphate (PO_4_^–3^; Td point group) Raman bands corresponding to symmetric stretch (v1) at 961 cm^−1^, bending (v2) at 431 & 454 cm^−1^, and bending (V4) at 581, 592 & 611 cm^−1^.

### Image adjustments, analysis, and presentation

Image processing and analysis were mostly performed in the native Carl Zeiss Zen (version 3.5) program used to acquire the images in the same system computer. Where needed the gamma of 0.45 or 0.75 was used under a spline mode where necessary to enhance the color and image fidelity for easy observation. Image enhancements were also made in the 3D rendering program Imaris, Canvas, Adobe Photoshop, Microsoft PowerPoint, when the final plates are cropped, resized and assembled.

### Ethics approval and consent to participate

This basic medical research study was reviewed and approved by the institutional review board of University of California at Los Angeles (IRB#19-000624).

## Results

### Aortic valve leaflets

**Figure 3:**
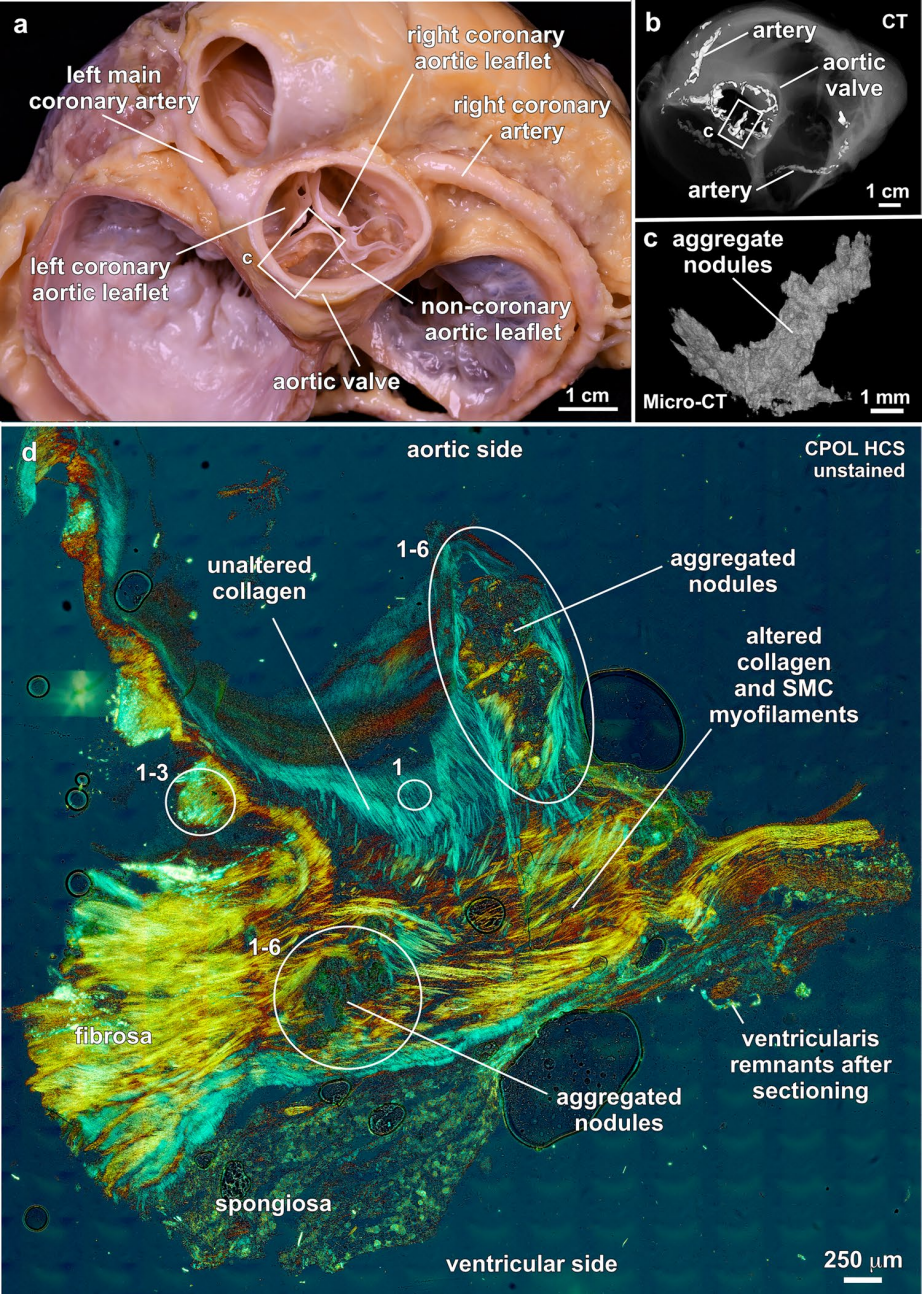
Physiological context and vertical histological cryosection of aortic valve leaflets. (**a**), Superior view of a dissected whole legacy heart. Large mm-scale aggregated ACP nodules grow within leaflet tissues (white box). (**b**), 600 μm-resolution CT scan corresponding to whole heart dissection shown in (**a**). Bright white areas indicate distribution of ACP deposits. (**c**), 3 μm-resolution Micro-CT scan of aggregate ACP nodules within the non-coronary aortic leaflet (white boxes in (**a**) and (**b**). (**d**), Simultaneous occurrence within the fibrosa tissues of unaltered collagen (blue birefringence), altered collagen and SMC myofilaments (yellow to red birefringence) and nodules (non-birefringent). Section prepared from area of white box in (**a**). Numbers refer to paragenetic sequence events in Fig. 2. CT = computed tomography. Micro-CT = micro-computed tomography. CPOL = circular polarization. HSC = histology cryosection.

Aortic valves contain left coronary, right coronary and non-coronary leaflets that develop large mm-scale calcified nodules within the leaflet tissues (Fig. 3a–c). Rather than passive structures involved in aortic valve function, each thin aortic valve leaflet (Fig. 3a) is instead a dynamic, innervated, muscled and vascularized organ with the capacity to adapt to complex cardiac environmental conditions and stresses^23,48,49^. Common examples of leaflet function include activation of myocyte and fibroblast differentiation during inflammatory response to calcification^50^, as well as ongoing adjustment of biomechanical properties through fibroblast-driven tissue remodeling in response to the hemodynamic forces constantly imparted over the three billion repetitive systole and diastole cycles of a human lifespan^23,48,49^. These adaptive structural and functional responses are made possible by communication between valvular endothelial cells (VECs) and valvular interstitial cells (VICs), which results in tissue remodeling and the synthesis of a valvular extracellular matrix (VECM), collagen and elastin^7,11,12,14,23,24,48,49,51–53^.

The outermost monolayer of VECs overlying and encasing each leaflet is central to aortic valve function, with individual cells oriented relative to blood flow direction, leaflet sheer stress and cytoskeletal structure^48^. VICs within leaflets contain a mixed population of cells dominated by interacting smooth muscle cells and fibroblasts^11,48,54^. The thin (300–700 μm-thick) yet extremely strong 3-layered structure of each leaflet, which have no initial calcification when healthy^23,48,49^, consists of (Fig. 3d): (1) a fibrosa layer on the aortic side (45% of leaflet thickness) that contains a mixed population of VICs and Type I collagen fibers^55–57^ for support against circumferential hemodynamic stresses and tensile forces imparted by aortic backflow during diastole; (2) a middle spongiosa layer (35% of leaflet thickness) with a microvascular system that permits interstitial cells to absorb shear forces during the cardiac cycle, an extracellular matrix that produces high concentrations of proteoglycan and glycosaminoglycan (GAG) and a high hydrous content for gliding of the layers during the cardiac cycles; and (3) a lowermost ventricularis layer (20% of leaflet thickness) that contains densely laminated and radially aligned elastin fibers that provide tissue structural flexibility, enhanced radial stretch and elastic recoil during systole. However, upon calcification and initiation of inflammatory responses, leaflets undergo complex modifications and remodeling^14^.

### Paragenetic sequence of aortic valve leaflet calcification events

**Figure 4:**
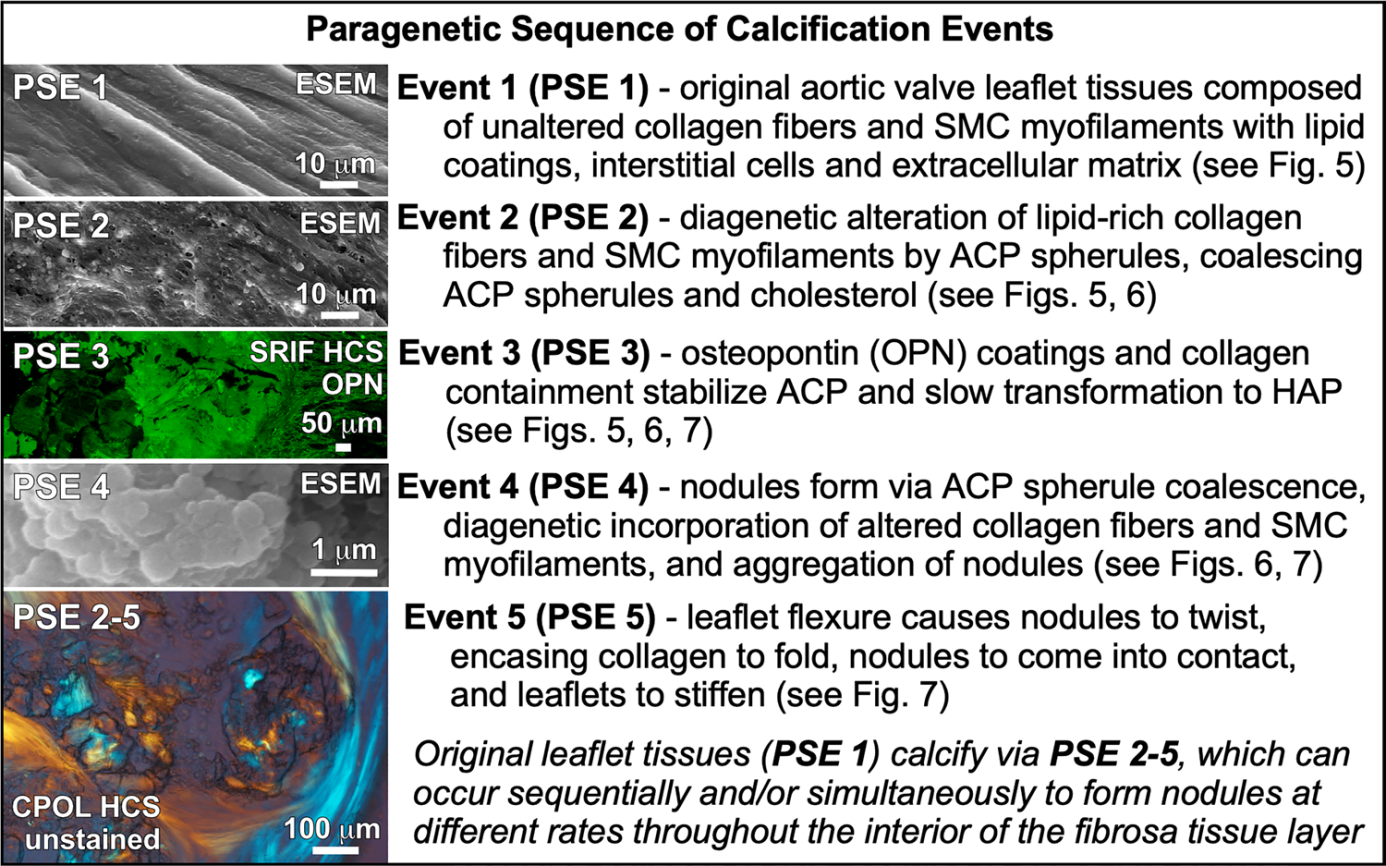
Paragenetic sequence of ACP and cholesterol calcification events within the fibrosa layer of human aortic valve leaflets. Each paragenetic sequence event (PSE) 1 though 5 are reconstructed from integrated multimodal microscopy and spectroscopy analyses (Table 1). Images of PSE 1 through 5 (left) are provided only as an initial example and are further described in detail in the Results section and Figs. 5, 6 and 7. ACP = amorphous calcium phosphate. ESEM = environmental scanning electron microscopy. SRIF = super resolution induced fluorescence. SMC = smooth muscle cell. HCS = histology cryosection. PSE = paragenetic sequence event.

**Figure 5:**
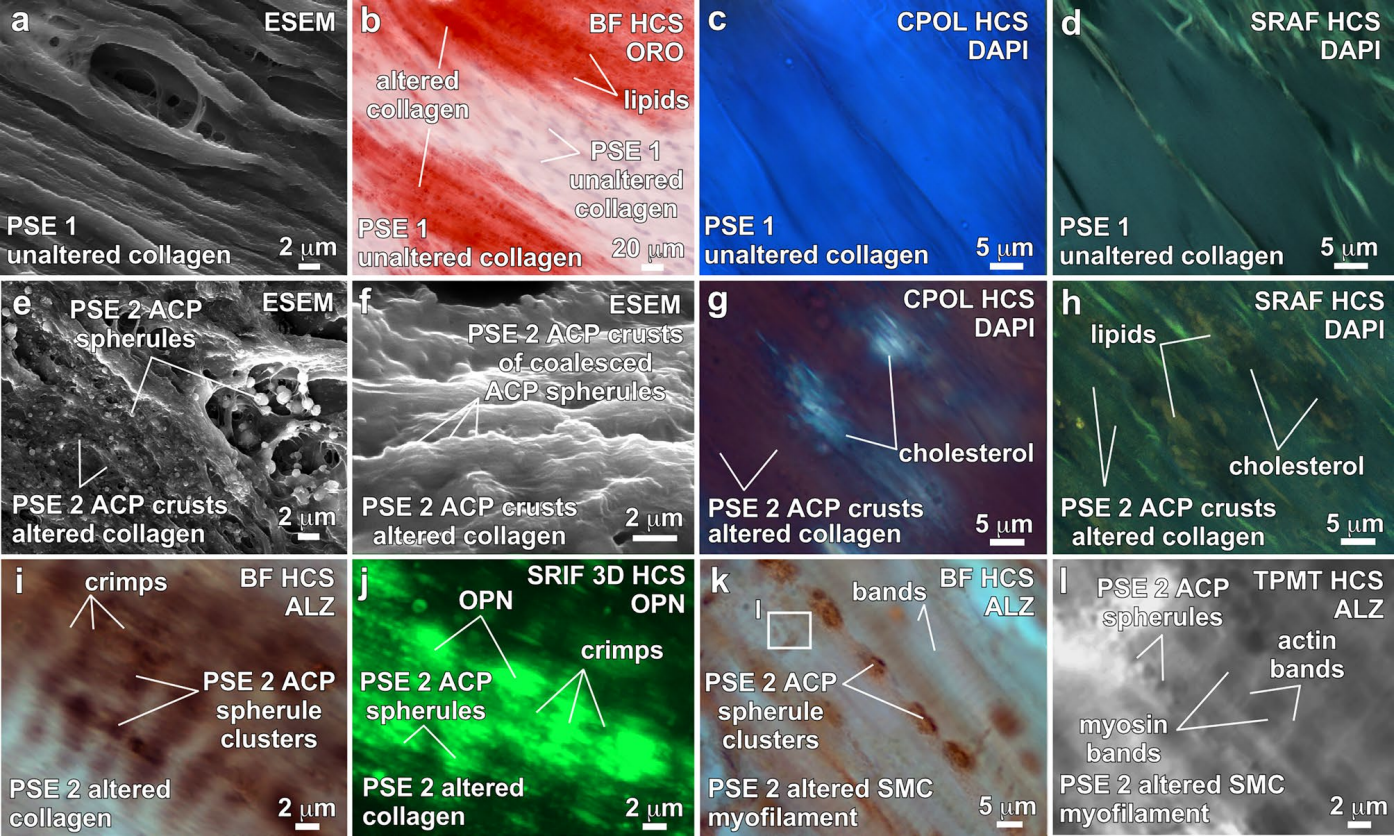
ACP and cholesterol calcification of collagen fibers and SMC myofilaments within the fibrosa tissue layer of human aortic valve leaflets. Integrated multimodal criteria used for ACP and cholesterol identification are summarized in Table 1. Modalities and stains used to collect each image are in the upper right and listed below. Complementary contextual imaging is presented in SI Figs. 2–5. Paragenetic sequence events (PSE 1–5) are described in Fig. 4. (**a**–**d**), Unaltered collagen fibers. (**b**) and (**e–h**), Altered collagen fibers and SMC myofilaments exhibiting ACP and cholesterol biomineralization. **i**, ACP spherule clusters (red stain) differentially distributed within crimped regions of altered collagen fibers. (**j**), OPN (green coatings) concentrated on ACP spherule clusters within crimped regions of altered collagen fibers. (**k**), ACP spherule clusters (red stain) regularly spaced within altered SMC myofilament banding. (**l**), Enlargement of white box in (**k**) showing a line of ACP spherules consistently spaced within altered SMC myofilament bands. ACP = amorphous calcium phosphate. SMC = smooth muscle cells. CPOL = circular polarization. HCS = histology cryosection. ESEM = environmental scanning electron microscope. SRAF = super resolution autofluorescence. SRIF = super resolution induced fluorescence. BF = bright field. TPMT = transmitted light photomultiplier tube. OPN = Alexa 647 antibody green stain for osteopontin. ALZ = Alizarin Red S stain for calcium. ORO = Oil Red O red stain for lipids. DAPI = 4′,6-diamidino-2-phenylindole stain for DNA.

Multiple independent lines of microscopy and spectroscopy evidence (Table 1) are combined here to establish a paragenetic sequence of ACP and cholesterol biomineralization events (Fig. 4) that result in the calcification of aortic valve leaflet tissues. All paragenetic sequence events (PSE 1–5; Fig. 4) are present simultaneously at different locations and at differential extents of development throughout each leaflet across a broad 10^7^ range of length scales. This is caused by the highly variable extents of reaction that occur at any one location^35^ as calcifying fluids permeate through the aortic valve leaflet tissues. CPOL extinction and birefringence are especially valuable tools for identifying and mapping calcification in unstained histological cryosections and petrographic sections (Fig. 3d). However, small changes in histological cryosection and petrographic section thickness and the axial orientation of collagen fibers can also influence CPOL birefringence^58^. As a result, confirmation of the presence and extent of collagen calcification (PSE 2; Fig. 4) implied by CPOL birefringence is substantiated in the present study with integrated multimodal analyses (Table 1). In original leaflet tissues (PSE 1; Fig. 4) the uppermost layers of the fibrosa layer are composed of unaltered collagen fibers (Fig. 3d) that contain no evidence of either ACP or cholesterol deposits as follows. Each unaltered collagen fiber exhibits low concentrations of lipid droplets in BF (Fig. 5b; SI Fig. 3a), blue CPOL birefringence (Figs. 3d, 5c; SI Fig. 4a,c), green SRAF emissions (Figs. 5d; SI Fig. 4a–c) and has no detectable Ca or P concentrations with EDAX analyses of serial cryosections (Table 1; Fig. 1; SI Fig. 1). These characteristics are consistent with previous reports of unaltered collagen birefringence created by its anisotropic fibrous structure^58,59^ and emissions from intrinsic biomolecules such as proteoglycans and glycosaminoglycans (GAG)^60,61^.

Medial and lower portions of the fibrosa tissue layer (Fig. 3d) exhibit variable extents of ACP and cholesterol diagenetic alteration of collagen fibers and smooth muscle cell (SMC) myofilaments (PSE 2; Fig. 4). This is indicated by their Raman spectra (Table 1; Fig. 2; SI Fig. 1), the presence of individual 100’s nm- to 1 μm-scale ACP spherules and crusts formed by coalescing ACP spherules in ESEM (Figs. 5e, f; SI Fig. 2d), EDAX compositional Ca/P atomic ratios of 1.17–2.49 ± 0.38 (Table 1; Fig. 1; SI Fig. 1), red to green to yellow CPOL birefringence (Figs. 3d, 5g; SI Fig. 3), yellow SRAF emissions (Fig. 5h; SI Fig. 4b), and low-contrast diffuse gray Micro-CT (Fig. 3c). 100’s nm-scale ACP spherules precipitate within the ultrastructure of crimped collagen fibers^57,62^ (Fig. 5i,j; SI Fig. 4e,f) and within the banded ultrastructure of SMC myofilaments on thick phosphorylated myosin bands, which are separated by thinner actin bands with less ACP precipitation (Fig. 5k,l)^50,63^. This combination of optical and electron microscopy and Raman spectroscopy evidence indicates the presence of multiple transient ACP diagenetic calcium phosphate phases based on their Ca/P atomic %^4,43^ (Table 1; Fig. 1). The poorly ordered transient composition^3^ of ACP spherules and nodules (Fig. 1) result in extinct black to 1st order dark gray lavender birefringence in CPOL (Table 1, Fig. 5g; SI Fig. 3c).

**Figure 6:**
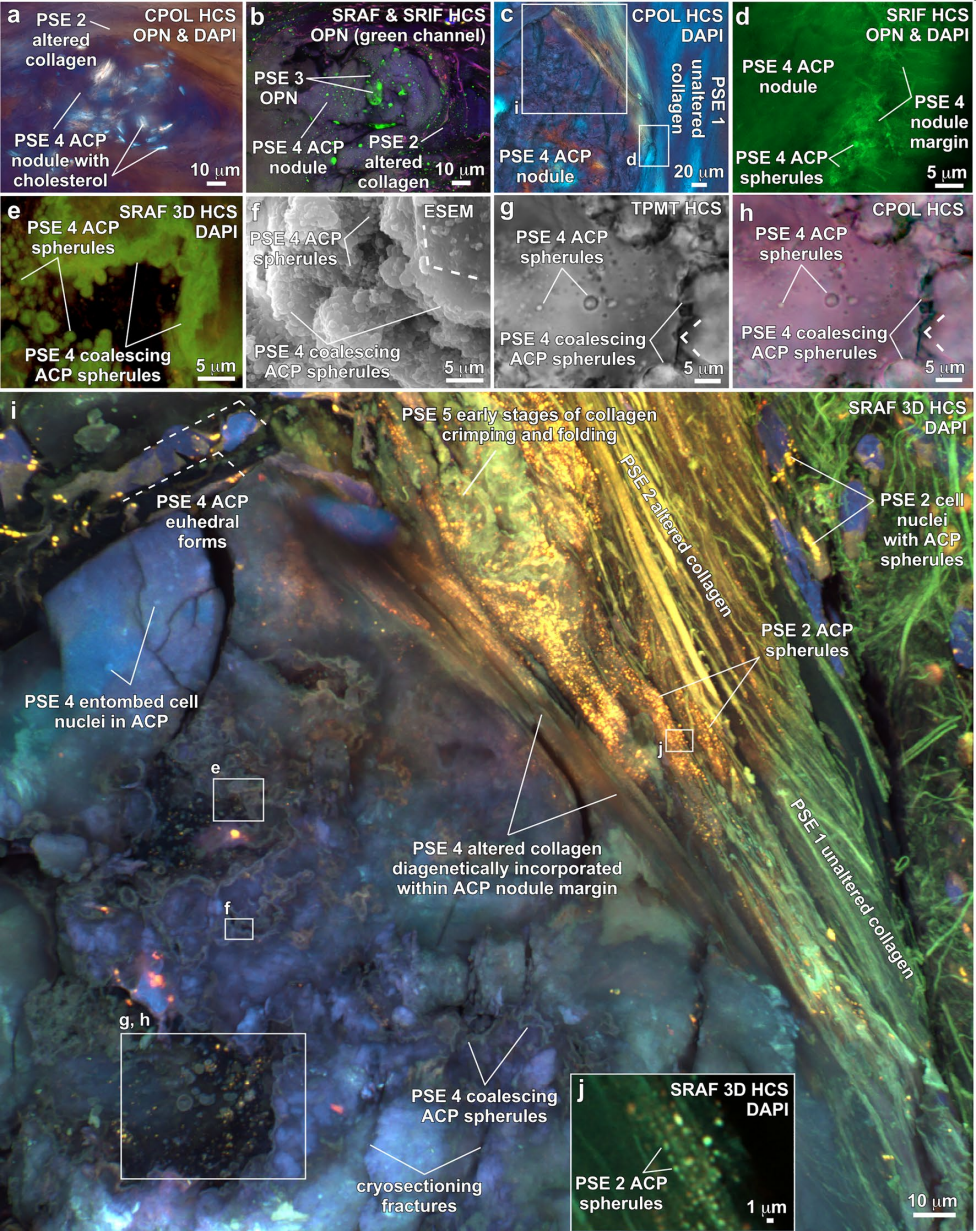
Nodule formation within the fibrosa layer of human aortic valve leaflets. Integrated multimodal criteria used for ACP and cholesterol identification are summarized in Table 1. Direct evidence for the 6-stage paragenetic sequence (PS) presented in Fig. 4 is shown in boxes labelled PS1 through 6. Modalities and stains used to collect each image are indicted in the upper right of each image and listed below. Complementary contextual imaging presented in SI Figs. 2, 4 and 6–9 and SI Movies 1–3. Paragenetic sequence events (PSE 1–5) are described in Fig. 4. (**a**) and (**b**), Paired images from a histological section (from areas in white box shown in Fig. 3a-c) oriented vertically through the leaflet that show early development of a nodule forming between and displacing altered collagen fibers. The nodule is composed of ACP and cholesterol biomineralization deposits that have OPN coatings (green immunofluorescence in (**b**). (**c**), Well-developed ACP nodule (shown in upper white oval in Fig. 3d) exhibiting low to absent birefringence, which is drapped and directly overlain by unaltered and altered collagen fibers. (**d**), Margin of an ACP nodule exhibiting concentrated OPN (green immunofluorescence). Enlargement of white box (**d**) shown in (**c**). (**e**), Autofluorescence of individual and coalescing ACP spherules in a nodule (enlargement of white box (**e**) shown in (**i**). (**f**), Individual and coalescing ACP spherules exhibit low order euhedral forms (white dashed lines). (**g**) and (**h**), Paired images of individual and coalescing ACP spherules within a nodule (enlargement of white box (**g**), (**h**) shown in (**i**). Coalescing ACP spherules exhibit low order euhedral forms (white dashed lines). **i**, Upper margin of an ACP nodule (mottled indigo to yellow autofluorescence) overlain by unaltered and altered collagen fibers (brown, red, green, and yellow autofluorescence). ACP nodule margins exhibit low order euhedral forms (white dashed lines) and is enlarging by diagenetically incorporating altered collagen fibers. (**j**), ACP spherules deposited at regular intervals within D-spacing ultrastructure bands of altered collagen fibers (enlargement of white box (**j**) shown in (**i**). ACP = amorphous calcium phosphate. CPOL = circular polarization. SRAF = super resolution autofluorescence. SRIF = super resolution induced fluorescence. 3D = three-dimensional. HCS = histology cryosection. BF = bright field. OPN = Alexa 647 antibody stain for osteopontin. DAPI = 4′,6-diamidino-2-phenylindole stain for DNA. ESEM = environmental scanning electron microscope.

ACP diagenetic alteration (PSE 2; Fig. 4) accentuates the CPOL blue birefringence of original collagen and SMC myofilament proteoglycans (SI Fig. 5) to create higher birefringence in areas of partial or complete calcification (Fig. 3d; SI Fig. 4a,c, 6b,f–h). Concentrated coatings of OPN observed on lipids, ACP and cholesterol (PSE 3; Figs. 4, 5j, 6d; SI Fig. 5) serve to chemically stabilize the ACP and prevent spontaneous transition to HAP^7,8,10,41^. In addition, the combination of Alizarin Red S staining and OPN coatings (analyzed on sequential histological cryosections) provide the contrast required to resolve: (1) Type I collagen fiber crimping (Fig. 5i,j; SI Fig. 4e,f) that is common in soft tissues and stores biomechanical energy during leaflet flexure^62^; and (2) ACP precipitation within the 1 μm-thick striated banding ultrastructure of SMC myofilaments (Fig. 5k,l; SI Fig. 5e,f)^64^. Cholesterol precipitates as parallel bundles of euhedral acicular crystals aligned between diagenetically altered collagen fibers and SMC myofilaments, as identified by their white CPOL birefringence (Fig. 5g; SI Fig. 3c), no SRAF emissions (Fig. 5h; SI Fig. 4c) and no detectable Ca or P concentrations with EDAX (Table 1). Furthermore, cholesterol crystals have previously been observed to enhance precipitation of 100’s nm-scale ACP spherules from synthetic cardiovascular fluids under controlled experimental conditions^37^.

**Figure 7:**
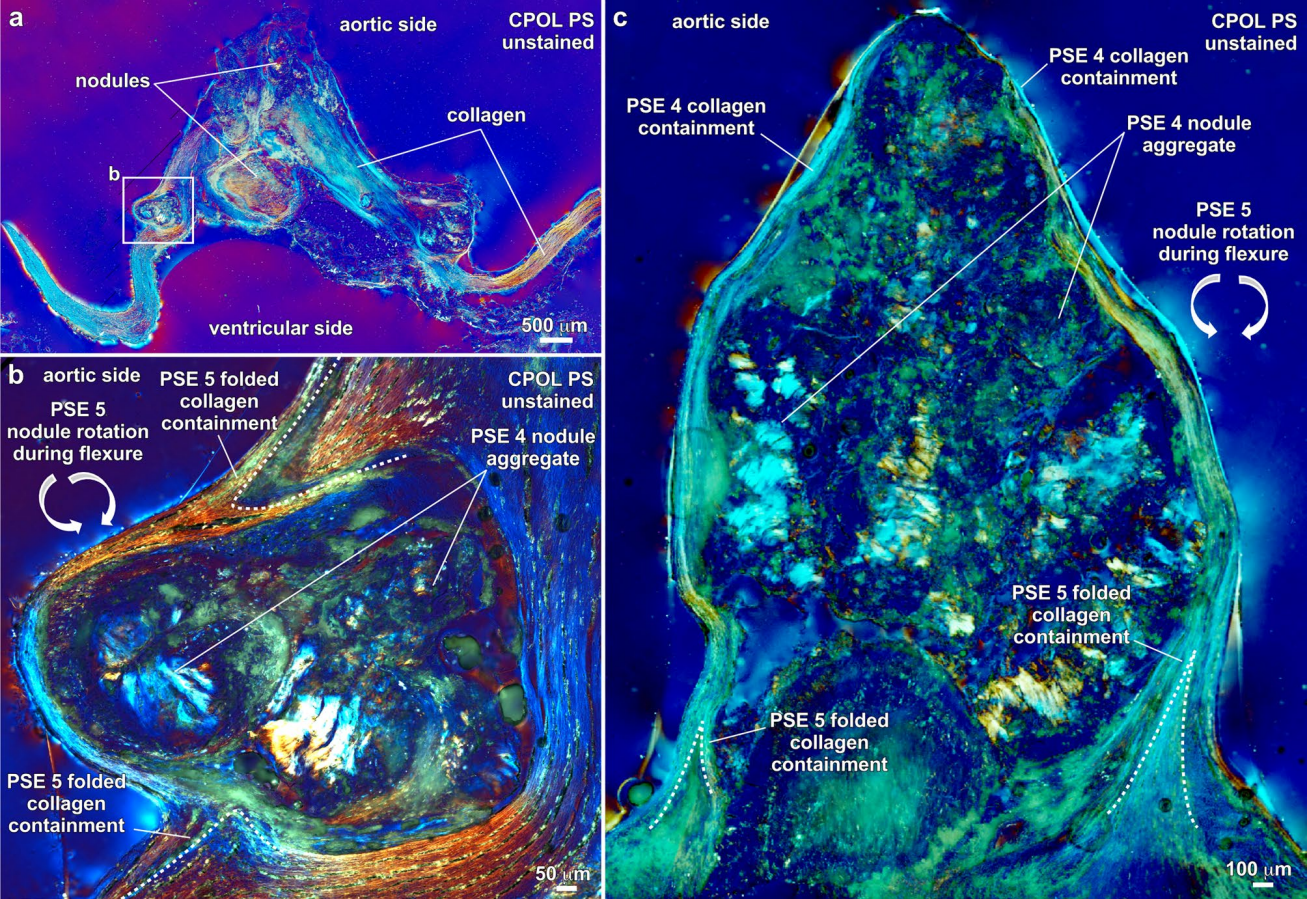
Containment of nodules by unaltered collagen fibers in the fibrosa tissue layer of human aortic valve leaflets. Integrated multimodal criteria used for ACP and cholesterol identification are summarized in Table 1a, b. Modalities and stains used to collect each image are in the upper right and listed below. Complementary contextual imaging presented in SI Figs. 8 and 9. Paragenetic sequence events (PSE 1–5) are described in Fig. 4. (**a**), Vertically oriented petrographic section showing collagen fibers (blue though yellow to red birefringence) surrounding aggregated ACP nodules (low birefringence) that occur in a variety of irregular bulbous shapes and sizes within the fibrosa tissue layer. (**b**) and (**c**), Aggregated ACP nodules forming at the outermost aortic margin of fibrosa tissues layers (white box in (**a**) for (**b**). Nodules are fully contained by unaltered collagen fibers and therefore protected from blood serum solution chemistry and water. Components of the aggregated nodules include ACP (dark red to green birefringence) and crystalline cholesterol (blue to white birefringence). ACP = amorphous calcium phosphate. PS = petrographic section. CPOL = circular polarization.

The presence of nodules (PSE 4; Fig. 4) is an emblematic feature of calcified aortic valve leaflet tissues^23,28,65^ (Fig. 6; SI Figs. 6–9). Evidence indicating that these nodules are primarily composed of ACP includes Raman spectra (Table 1; Fig. 2; SI Fig. 1), low contrast diffuse gray Micro-CT (Fig. 3c), extinct black to dark gray lavender CPOL birefringence (Figs. 3d, 6a, c, h), yellow to red to indigo SRAF (Fig. 6e, i, j; SI Figs. 6–9), and EDAX Ca/P atomic ratios of 1.47–1.91 (Table 1; Fig. 1; SI Fig. 1). In addition, ACP and cholesterol nodules of all sizes are coated with OPN (Figs. 6b, d; SI Fig. 6b, c, f–h). The cores of nodules formed by coalescing ACP spherules commonly exhibit rudimentary euhedral geometric forms^11^ (Figs. 6f-i) that are similar to transformations observed in human kidney stones and natural hot springs^6^. Simultaneously, the outermost nodule margins diagenetically incorporate surrounding ACP altered collagen fibers via Ångstrom-scale fabric preserving (*mimetic*) dissolution and reprecipitation replacement^6^ (Fig. 6i; SI Movies 1–3). Importantly, all diagenetic paragenetic sequence events (PSE 2–5; Fig. 4) are observed to occur exclusively within, and not on the VECs and outermost surface of the aortic side of the fibrosa tissue layer (Fig. 3d; SI Figs. 8, 9). Nodules range from small 10’s μm-diameter single deposits growing between collagen fibers (Figs. 6a, b; SI Fig. 6) to large well-developed mm-sized aggregate nodules (Figs. 3d, 6i; SI Figs. 7–9). Initially, small nodules are composed of approximately equivalent amounts of ACP and acicular cholesterol crystals (Figs. 6a, b). Conversely, as nodule enlargement progresses through intermediate and advanced stages of calcification (PSE 4; Fig. 4), nodules become predominantly composed of ACP with significantly lower amounts of cholesterol (Figs. 6c–i, 7; SI Figs. 7–9).

**Figure 8:**
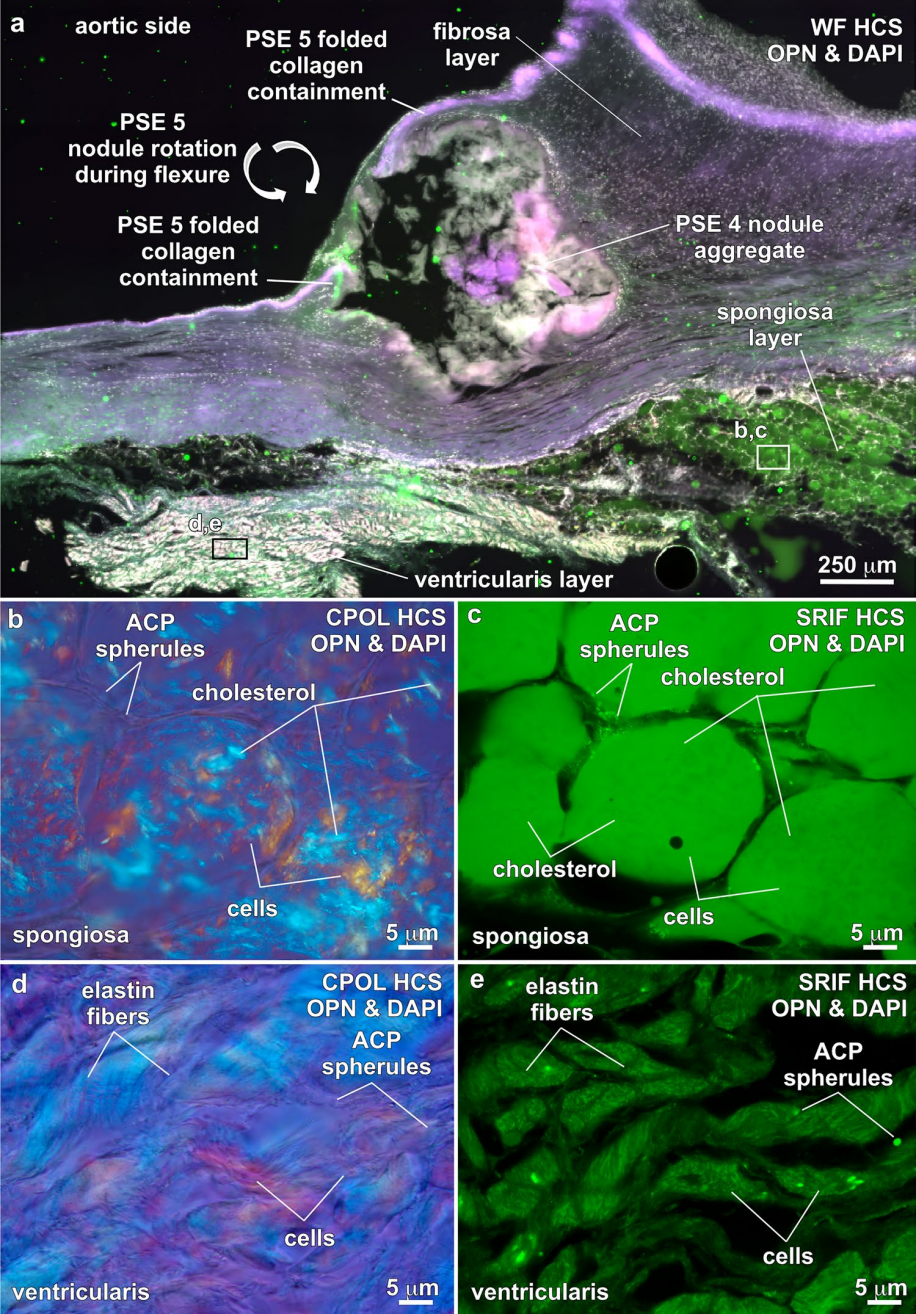
ACP and cholesterol biomineralization within the spongiosa and ventricularis layers of human aortic valve leaflets. Integrated multimodal criteria used for ACP and cholesterol identification are summarized in Table 1a, b. Modalities and stains used to collect each image are in the upper right and listed below. Paragenetic sequence events (PSE 1–5) are described in Fig. 4. (**a**) Fibrosa, spongiosa and ventricularis layers. Nodule at the outermost margin of the fibrosa layer exhibits collagen folding (from rotation) and collagen containment. (**b** and **c**), Spongiosa layer (from areas in white box in Fig. 1a-c) showing extensive cholesterol crystallization and minimal precipitation of ACP spherules exclusively at the interface of the peripheral cell cytoplasm and membrane. (**d** and **e**), Ventricularis layer showing the presence of elastin and the absence of cholesterol biomineralization. Rare ACP spherules occur within cell cytoplasm. ACP = amorphous calcium phosphate. WF = widefield. HCS = histology cryosection. OPN = Alexa 647 antibody stain for osteopontin. DAPI = 4′,6-diamidino-2-phenylindole stain for DNA. CPOL = circular polarization. SRIF = super resolution induced fluorescence.

### Nodule twists, stenosis and containment

Calcification nodules (PSE 4; Fig. 4) form everywhere from deep within the leaflet fibrosa tissue layer to near the aortic margin and are overlain by 10’s μm-thick continuous layers of unaltered and unbroken collagen fibers (Figs. 3d, 6c,i, 7; SI Figs. 6–9). The interior underside of these collagen bundles, which are in direct proximity with each nodule, stretch during leaflet flexure and become increasingly more altered until becoming diagenetically incorporated into the nodule outer surfaces, allowing nodules to grow, expand and accrete (Figs. 6i, 7; SI Figs. 8, 9). However, an outermost containment barrier of unaltered and unbroken collagen is simultaneously maintained that prevents nodules from rupturing and penetrating the outermost VECs and fibrosa tissues (Fig. 7; SI Figs. 7–9). This containment mechanism prevents the ACP-dominated nodules from being directly exposed to blood solution chemistry (pH, saturation state, and availability of H_2_O). Exposure and direct contact with ACP-saturated and H_2_O-rich blood serum would quickly drive transformation of ACP into HAP and rapidly increase the extent and distribution of calcification^4,66^. A similar type of Type 1 collagen containment process encapsulates, restrains and decelerates the progression of pancreatic ductal adenocarcinoma cancer tumors^67^.

Stress and strain forces during each diastole and systole cycle induce leaflet tissue flexure^68^, which in turn causes the hard calcified nodules to continually rotate back-and-forth (twist) as they are forming (PSE 5; Fig. 4). This process is recorded by asymmetrically folded and overfolded^69^ unaltered collagen fibers that surround and contain small-to-large single and aggregated nodules (Figs. 6i, 7b, c, 8a; SI Figs. 8, 9). These folds store, transmit and dissipate elastic energy^56^ as surrounding collagen axially extends and contracts to prevent the fibers from tearing and breaking during twisting rotational motions of the nodules during leaflet flexure^70^. As additional nodules form in the fibrosa layer with increasing extents of calcification, nodules further twist and turn, physically contact each other, and eventually aggregate, which dramatically increases the elastic modulus of the tissue to cause stiffening and further reduce leaflet structural flexibility and function. A similar mechanism occurs during water motion-induced flexure of pliable connective tissues in marine benthic invertebrates such as sponges and soft corals, which causes spatially isolated mm-scale tissue hard parts (spicules) to contact each other, amplify strain, resist compression and increase elastic modulus and stiffness^71^.

### Spongiosa and ventricularis ACP and cholesterol biomineralization

Unlike the dominance of fibrosa tissue layer ACP diagenetic alteration in the form of altered collagen, altered SMC myofilaments, and nodules, the lipid-rich spongiosa layer (Fig. 8a) is predominantly altered by acicular cholesterol crystals that form in cell cytoplasm. These clusters of acicular cholesterol crystals exhibit white to blue CPOL birefringence (Fig. 8b) and highly concentrated coatings of OPN (Fig. 8c). ACP biomineralization is minimal in the spongiosa, forming rare individual spherules at peripheral cell cytoplasm and membrane interfaces that exhibit a dark lavender gray CPOL, yellow SRAF emissions and have pervasive OPN coatings (Fig. 8c). In contrast, elastin-dense ventricularis tissues contain no cholesterol crystals (Fig. 8d) and exhibit only rare occurrences of ACP spherules (dark gray lavender CPOL and yellow SRAF emissions) within cell cytoplasm (Fig. 8e). The heterogeneously distributed moderate to high concentrations of OPN observed throughout the ventricularis layer (Figs. 8e) is consistent with previous observations^72^. Despite extensive cholesterol crystallization, some ACP spherules and moderate to high concentrations of lipids and OPN, there is no evidence of ACP nodule formation in the spongiosa and ventricularis cell layers as there is in the fibrosa layer.

## Discussion

### Implications for translational medicine

The paragenetic sequence of ACP and cholesterol biomineralization observed here (PSE 2–5; Fig. 4), which has been tracked across 10’s nm to 10’s cm length-scales within dissected samples and tissue sections, provides independent evidence to test current assumptions regarding mechanisms of aortic valve leaflet calcification. Previous cellular and molecular pathophysiology studies propose that calcification is initiated by external damage and scarring of the VEC and outermost fibrosa layer due to oxidative and biomechanical stress^7,11–15,23,24^. In this widely held scenario, ACP-saturated and water-rich blood serum solutions containing lipoproteins and immune cells would infiltrate through these damaged breeches, in a manner similar to that of saturated urine in the thin loops of Henle during human kidney stone formation^73^. Resulting inflammation would activate reactive oxygen species, monocytes and macrophages (multinucleated giant cells) and cause VICs to differentiate into fibroblast, osteoblast-like and SMC myofilament phenotypes that promote fibrosis, calcification, matrix remodeling and leaflet stenosis. Ensuing chronic inflammation would then activate macrophage and VIC apoptosis, with the release of Ca, P and extracellular vesicles further promoting a self-perpetuating cycle of calcification primarily at locations near aortic side breeches of the fibrosa layer of the leaflet tissues.

However, ACP and cholesterol biomineralization observed in the present study exhibits no evidence of calcification on the outer surface of the leaflets and no evidence of damage or breech of the VEC or outermost fibrosa tissues (Fig. 7; SI Figs. 8, 9). Instead, all diagenetic stages of the paragenetic sequence (PSE 2–5; Fig. 4) and small-to-large nodules that occur throughout the fibrosa tissues, with nodules surrounded and contained by continuous, tightly stretched, and unbroken outermost layers of non-calcified collagen fibers (Figs. 3d, 7; SI Figs. 8, 9). These in vivo mechanisms create the commonly observed bulbous appearance of calcified aortic valve leaflets^23,65^ (Fig. 3a; SI Fig. 2a, b; 7–9). The GeoBioMed evidence collected in the present study implies that calcification can take place without obvious damage of the outermost VEC and fibrosa tissues. As a result, original leaflet tissue permeability may be sufficient to permit the ongoing normally occurring infiltration of saturated lipid-rich serum-derived solutions that are capable of in vivo calcification. In addition to these permeating fluids, ACP-rich vesicles and lipids may also be delivered inside the leaflet tissues via microvasculature^14^.

Future GeoBioMed research will focus on the mechanisms of OPN stabilization and collagen containment to slow, prevent and disrupt key calcification events of the paragenetic sequence within aortic valve leaflet fibrosa tissues (Fig. 4). Targets include nm-scale ACP spherule precipitation, Ostwald ripening and coalescence of spherules into planar forms, and diagenetic recrystallization and incorporation of altered collagen at the margin of nodules. This will require further strategic multimodal analyses of calcified aortic valve leaflets from patients with replaced aortic valves and prosthetic implants^10^. The banding-specific deposition of ACP spherules within collagen crimps (Figs. 5i, j; SI Figs. 4e, f) and SMC myofilaments (Figs. 5k, l; SI Figs. 5e, f), suggests that ACP precipitation is substrate controlled by long-axis parallel and perpendicular ultrastructure and compositional changes within collagen fibers^57,74,75^ and SMC myofilaments^76^. These types of individual and combined mechanisms controlling the paragenetic sequence of calcification events (Fig. 4), which combine to prevent reliable thermodynamic predictions, can now be systematically tested in future studies using controlled microfluidic experimentation^77^. Previous experimental studies using blood serum and other fluids indicate that the initiation and thermodynamic stabilization of ACP is primarily controlled by^8,10^: (1) solution organic and inorganic chemistry and flow regime (e.g., pH; temperature; alkalinity; concentrations of Ca, P, Mg, Zn; organic molecule concentrations such as ATP, poly-l-lysine/citrate and poly-Asp, and boundary layer diffusion); (2) availability, concentration and distribution of proteins, phosphopeptides and other complexes that stabilize ACP and prevent or promote transition to HAP (e.g., phosphorylated OPN, casein, Mg); and (3) structure and composition of the substrate of precipitation. As each of these influences on ACP precipitation are systematically varied, the resulting composition, distribution and rate of ACP and cholesterol biomineralization can be tracked spatially and temporally in real time within microfluidic test beds such as the GeoBioCell^78^. These processes can be quantitatively tracked during systematic changes in aortic valve hydrology^47^ and ACP distribution and composition (e.g., elemental, isotopic, structural, trapped biomolecules) in the presence of hydrogels embedded with living VICs, collagen and SMC myofilaments^77^. Another complimentary and closely coordinated approach would be to experimentally track the precipitation of 100’s nm- to 1 μm-diameter ACP spherules within collagen crimps (Fig. 5i, j; SI Figs.4e, f) and SMC myofilaments (Figs. 5k, l; SI Figs. 5e, f). Controlled microfluidic experimentation would also permit testing of potential dosing effects of proteins such as OPN and nutraceuticals including Mg, Zn, Fe, vitamin K, phytate and natural plant derived compounds such as curcumin^79^. Collectively, these types GeoBioMed analytical approaches and experimentation, guided by the paragenetic sequence (Fig. 4), will permit discovery of fundamentally new approaches for the development of clinical therapies targeting the prevention and treatment of aortic valve leaflet calcification.

### Supplementary Information

Supplementary Figures.

Supplementary Video 1.

Supplementary Video 2.

Supplementary Video 3.

## Data Availability

Raw microscope images and processed images are available for download from the following link: https://figshare.com/s/c8ab95abe42b4d65d971

## References

[1] Lowenstam HA, Weiner S (1985). Transformation of amorphous calcium phosphate to crystalline dahllite in the radular teeth of chitons. Science.

[2] Pulletikurti S, Veena KS, Yadav M, Deniz AA, Krishnamurthy R (2024). Experimentally modeling the emergence of prebiotically plausible phospholipid vesicles. Chem.

[3] Degli Esposti L, Iafisco M (2022). Amorphous calcium phosphate, the lack of order is an abundance of possibilities. Biomater. Biosyst..

[4] Dorozhkin S (2009). Calcium orthophosphates in nature, biology and medicine. Materials.

[5] Habraken WJEM (2013). Ion-association complexes unite classical and non-classical theories for the biomimetic nucleation of calcium phosphate. Nat. Commun..

[6] Sivaguru M (2021). Human kidney stones: a natural record of universal biomineralization. Nat. Rev. Urol..

[7] Chen J-H, Simmons CA, Towler DA (2011). Cell-matrix interactions in the pathobiology of calcific aortic valve disease. Circul. Res..

[8] Holt C, Lenton S, Nylander T, Sørensen ES, Teixeira SCM (2014). Mineralisation of soft and hard tissues and the stability of biofluids. J. Struct. Biol..

[9] Ibsen CJS, Gebauer D, Birkedal H (2016). Osteopontin stabilizes metastable states prior to nucleation during apatite formation. Chem. Mater..

[10] Schweikle M (2019). Stabilisation of amorphous calcium phosphate in polyethylene glycol hydrogels. Acta Biomater..

[11] Broeders W (2022). Innate immune cells in the pathophysiology of calcific aortic valve disease: Lessons to be learned from atherosclerotic cardiovascular disease?. Basic Res. Cardiol..

[12] Moncla L-HM, Briend M, Bossé Y, Mathieu P (2023). Calcific aortic valve disease: mechanisms, prevention and treatment. Nat. Rev. Cardiol..

[13] Perez KA, Deppe DW, Filas A, Singh SA, Aikawa E (2023). Multimodal analytical tools to enhance mechanistic understanding of aortic valve calcification. Am. J. Pathol..

[14] Anousakis-Vlachochristou N, Athanasiadou D, Carneiro KMM, Toutouzas K (2023). Focusing on the native matrix proteins in calcific aortic valve stenosis. JACC Basic Trans. Sci..

[15] Sud K (2023). The contribution of amyloid deposition in the aortic valve to calcification and aortic stenosis. Nat Rev. Cardiol..

[16] Alyesh DM (2019). Postinfarction myocardial calcifications on cardiac computed tomography. Circul. Arrhythmia Electrophysiol..

[17] Thompson RC (2013). Atherosclerosis across 4000 years of human history: The Horus study of four ancient populations. The Lancet.

[18] Wells FC (2013). The Heart of Leonardo: Foreword by HRH Prince Charles, The Prince of Wales.

[19] Rafiee MJ, Bandegi P, Taylor JL (2024). Extensive myocardial calcifications in a dialysis patient: A porcelain heart manifesting with abdominal pain. Radiol. Case Rep..

[20] Radvar E (2021). Engineered in vitro models for pathological calcification: routes toward mechanistic understanding. Adv. NanoBiomed. Res..

[21] Sutton NR (2023). Molecular mechanisms of vascular health: insights from vascular aging and calcification. ATVB.

[22] Curini L, Pesce M (2023). Shockwaves delivery for aortic valve therapy—Realistic perspective for clinical translation?. Front. Cardiovasc. Med.

[23] Aikawa, E. & Schoen, F. J. Calcific and Degenerative Heart Valve Disease. in *Cellular and Molecular Pathobiology of Cardiovascular Disease* 161–180 (Elsevier, 2014). 10.1016/B978-0-12-405206-2.00009-0.

[24] Balachandran K, Sucosky P, Yoganathan AP (2011). Hemodynamics and mechanobiology of aortic valve inflammation and calcification. Int. J. Inflam..

[25] Proudfoot D (2019). Calcium signaling and tissue calcification. Cold Spring. Harb. Perspect. Biol..

[26] Kim KM, Trump BF (1975). Amorphous calcium precipitations in human aortic valve. Calc. Tis. Res..

[27] Roijers RB (2008). Early calcifications in human coronary arteries as determined with a proton microprobe. Anal. Chem..

[28] Cottignoli V, Cavarretta E, Salvador L, Valfré C, Maras A (2015). Morphological and chemical study of pathological deposits in human aortic and mitral valve stenosis: A biomineralogical contribution. Pathol. Res. Int..

[29] Bertazzo S, Gentleman E (2017). Aortic valve calcification: A bone of contention. Eur. Heart J..

[30] Weiner S, Addadi L (2011). Crystallization pathways in biomineralization. Ann. Rev. Mater. Res..

[31] Lovett AC, Khan SR, Gower LB (2019). Development of a two-stage in vitro model system to investigate the mineralization mechanisms involved in idiopathic stone formation: Stage 1-biomimetic Randall’s plaque using decellularized porcine kidneys. Urolithiasis.

[32] Zhao J, Liu Y, Sun W, Yang X (2012). First detection, characterization, and application of amorphous calcium phosphate in dentistry. J. Dental Sci..

[33] Boonrungsiman S (2012). The role of intracellular calcium phosphate in osteoblast-mediated bone apatite formation. Proc. Nat. Acad. Sci..

[34] Wegst UGK, Bai H, Saiz E, Tomsia AP, Ritchie RO (2015). Bioinspired structural materials. Nat. Mater..

[35] Banner JL, Hanson GN (1990). Calculation of simultaneous isotopic and trace element variations during water-rock interaction with applications to carbonate diagenesis. Geochimica et Cosmochimica Acta.

[36] Mahamid J, Sharir A, Addadi L, Weiner S (2008). Amorphous calcium phosphate is a major component of the forming fin bones of zebrafish: Indications for an amorphous precursor phase. Proc. Natl. Acad. Sci. USA..

[37] Dorozhkin SV, Dorozhkina EI (2005). In vitro simulation of vascular calcification by the controlled crystallization of amorphous calcium phosphates onto porous cholesterol. J. Mater. Sci..

[38] Gelli R, Ridi F, Baglioni P (2019). The importance of being amorphous: calcium and magnesium phosphates in the human body. Adv. Coll. Interface Sci..

[39] Gower LB (2008). Biomimetic model systems for investigating the amorphous precursor pathway and its role in biomineralization. Chem. Rev..

[40] Gower L, Elias J (2022). Colloid assembly and transformation (CAT): The relationship of PILP to biomineralization. J. Struct. Biol. X.

[41] Rodriguez DE (2014). Multifunctional role of osteopontin in directing intrafibrillar mineralization of collagen and activation of osteoclasts. Acta Biomaterialia..

[42] Montes-Hernandez G, Renard F (2020). Nucleation of brushite and hydroxyapatite from amorphous calcium phosphate phases revealed by dynamic *in situ* raman spectroscopy. J. Phys. Chem. C.

[43] Stammeier JA, Purgstaller B, Hippler D, Mavromatis V, Dietzel M (2018). In-situ Raman spectroscopy of amorphous calcium phosphate to crystalline hydroxyapatite transformation. MethodsX.

[44] Sainger R (2012). Dephosphorylation of circulating human osteopontin correlates with severe valvular calcification in patients with calcific aortic valve disease. Biomarkers.

[45] Gericke A (2005). Importance of phosphorylation for osteopontin regulation of biomineralization. Calcif. Tissue Int..

[46] Park JJ, Roudier MP, Soman D, Mokadam NA, Simkin PA (2014). Prevalence of birefringent crystals in cardiac and prostatic tissues, an observational study. BMJ Open.

[47] Sivaguru M (2018). Geobiology reveals how human kidney stones dissolve in vivo. Sci. Rep..

[48] Chester AH (2014). The living aortic valve: From molecules to function. Glob. Cardiol. Sci. Pract..

[49] Wang Y-W, Christenson HK, Meldrum FC (2014). Confinement increases the lifetimes of hydroxyapatite precursors. Chem. Mater..

[50] De Villiers JA, Houreld N, Abrahamse H (2009). Adipose derived stem cells and smooth muscle cells: Implications for regenerative medicine. Stem Cell. Rev. Rep..

[51] Mori S, Shivkumar K (2021). Real three-dimensional cardiac imaging using leading-edge holographic display. Clin. Anatomy.

[52] Rodriguez KJ, Piechura LM, Porras AM, Masters KS (2014). Manipulation of valve composition to elucidate the role of collagen in aortic valve calcification. BMC Cardiovas. Disorders.

[53] Müller KH (2019). Poly(ADP-Ribose) links the DNA damage response and biomineralization. Cell Rep..

[54] Baum J, Duffy HS (2011). Fibroblasts and myofibroblasts: What are we talking about?. J. Cardiovas. Pharmacol..

[55] Fang M, Holl MMB (2013). Variation in type I collagen fibril nanomorphology: the significance and origin. Bonekey Rep..

[56] *Collagen: Structure and Mechanics*. (Springer, New York, 2008).

[57] Lin N, Liu XY (2015). Correlation between hierarchical structure of crystal networks and macroscopic performance of mesoscopic soft materials and engineering principles. Chem. Soc. Rev..

[58] Spiesz EM, Kaminsky W, Zysset PK (2011). A quantitative collagen fibers orientation assessment using birefringence measurements: Calibration and application to human osteons. J. Struct. Biol..

[59] Lien C-H, Chen Z-H, Phan Q-H (2022). Birefringence effect studies of collagen formed by nonenzymatic glycation using dual-retarder Mueller polarimetry. J. Biomed. Opt..

[60] Croce AC, Bottiroli G (2014). Autofluorescence spectroscopy and imaging: A tool for biomedical research and diagnosis. Eur. J Histochem..

[61] Stephens CJ, Ladden SF, Meldrum FC, Christenson HK (2010). Amorphous calcium carbonate is stabilized in confinement. Adv. Funct. Mater..

[62] Spiesz EM, Thorpe CT, Thurner PJ, Screen HRC (2018). Structure and collagen crimp patterns of functionally distinct equine tendons, revealed by quantitative polarised light microscopy (qPLM). Acta Biomater..

[63] Latif N, Sarathchandra P, Chester AH, Yacoub MH (2015). Expression of smooth muscle cell markers and co-activators in calcified aortic valves. Eur. Heart J..

[64] Camelliti P, Borg TK, Kohl P (2005). Structural and functional characterisation of cardiac fibroblasts. Cardiovas. Res..

[65] Fletcher AJ, Singh T, Syed MBJ, Dweck MR (2021). Imaging aortic valve calcification: significance, approach and implications. Clin. Radiol..

[66] Delgado-López JM (2017). The synergic role of collagen and citrate in stabilizing amorphous calcium phosphate precursors with platy morphology. Acta Biomater..

[67] Chen Y (2021). Type I collagen deletion in αSMA+ myofibroblasts augments immune suppression and accelerates progression of pancreatic cancer. Cancer Cell.

[68] Zebhi B, Lazkani M, Bark D (2021). Calcific aortic stenosis—A review on acquired mechanisms of the disease and treatments. Front. Cardiovasc. Med..

[69] Nabavi ST, Fossen H (2021). Fold geometry and folding—A review. Earth-Sci. Rev..

[70] Bielajew BJ, Hu JC, Athanasiou KA (2020). Collagen: Quantification, biomechanics and role of minor subtypes in cartilage. Nat. Rev. Mater..

[71] Wainwright SA, Biggs WD, Currey JD, Gosline JM (1982). Mechanical design in organisms.

[72] O’Brien KD (1995). Osteopontin is expressed in human aortic valvular lesions. Circulation.

[73] Sivaguru M, Fouke BW (2022). Renal macrophages and multinucleated giant cells: Ferrymen of the river styx?. Kidney.

[74] Landis WJ, Song MJ, Leith A, McEwen L, McEwen BF (1993). Mineral and organic matrix interaction in normally calcifying tendon visualized in three dimensions by high-voltage electron microscopic tomography and graphic image reconstruction. J. Struct. Biol..

[75] Lotsari A, Rajasekharan AK, Halvarsson M, Andersson M (2018). Transformation of amorphous calcium phosphate to bone-like apatite. Nat. Commun..

[76] Sivaguru M (2010). Quantitative analysis of collagen fiber organization in injured tendons using Fourier transform-second harmonic generation imaging. Opt. Express OE.

[77] Mendoza M, Chen M-H, Huang P, Mahler GJ (2022). Shear and endothelial induced late-stage calcific aortic valve disease-on-a-chip develops calcium phosphate mineralizations. Lab. Chip..

[78] Fouke BW (2022). Sulfate-reducing bacteria streamers and iron sulfides abruptly occlude porosity and increase hydraulic resistance in proppant-filled shale fractures. Bulletin.

[79] Donato M (2021). The emerging role of nutraceuticals in cardiovascular calcification: Evidence from preclinical and clinical studies. Nutrients.

