# Supplementary material for: Osteopontin stabilization and collagen containment slows amorphous calcium phosphate transformation during human aortic valve leaflet calcification
Source: Sci Rep. 2024 May 28;14:12222. doi: 10.1038/s41598-024-62962-8 (PMC11133482; doi:10.1038/s41598-024-62962-8)
Supplement: Supplementary file 1 — Supplementary Figures. [file 41598_2024_62962_MOESM1_ESM.pdf]

Osteopontin stabilization and collagen containment slows amorphous calcium phosphate transformation during human aortic valve leaflet calcification

Mayandi Sivaguru[1,2*], Shumpei Mori[3], Kyle W. Fouke[4], Olujimi A. Ajijola[3], Kalyanam Shivkumar[3], Ashok Zachariah Samuel[5,6], Rohit Bhargava[5,6,7,8] and Bruce W. Fouke[2,9,10,11*]

1Cytometry and Microscopy to Omics Facility, Roy J. Carver Biotechnology Center, University of Illinois at Urbana-Champaign, Urbana, IL, USA.

2Earth Science & Environmental Change, School of Earth, Society and the Environment, University of Illinois at Urbana-Champaign, Urbana, IL, USA.

3Cardiac Arrhythmia Center and Neurocardiology Research Program of Excellence, David Geffen School of Medicine, UCLA Health, University of California Los Angeles, Los Angeles, CA, USA.

4Department of Earth and Planetary Sciences, Jackson School of Geosciences, University of Texas at Austin, Austin, TX, USA.

5Department of Bioengineering, Grainger College of Engineering, University of Illinois at Urbana-Champaign, Urbana, IL, USA.

6Beckman Institute for Advanced Science and Technology, University of Illinois at Urbana-Champaign, Urbana, IL, USA.

7Department of Chemical and Biological Engineering, Grainger College of Engineering, University of Illinois at Urbana-Champaign, Urbana, IL, USA.

8Cancer Center at Illinois, University of Illinois at Urbana-Champaign, Urbana, IL, USA.

9Biomedical and Translational Sciences, Carle Illinois College of Medicine, University of Illinois at Urbana-Champaign, Urbana, IL, USA.

10Department of Evolution, Ecology and Behavior, School of Integrative Biology, University of Illinois at Urbana-Champaign, Urbana, IL, USA.

11Roy J. Carver Biotechnology Center, University of Illinois at Urbana-Champaign, Urbana, IL, USA.

*Corresponding Authors: sivaguru@illinois.edu, fouke@illinois.edu

Keywords: amorphous calcium phosphate (ACP); hydroxyapatite (HAP); aortic valve; cardiovascular calcification; coalescing spherules; nodules; collagen alteration; collagen containment; cholesterol; GeoBioMed; lipids; osteopontin; spherules; super-resolution autofluorescence

Supplementary Data Figures

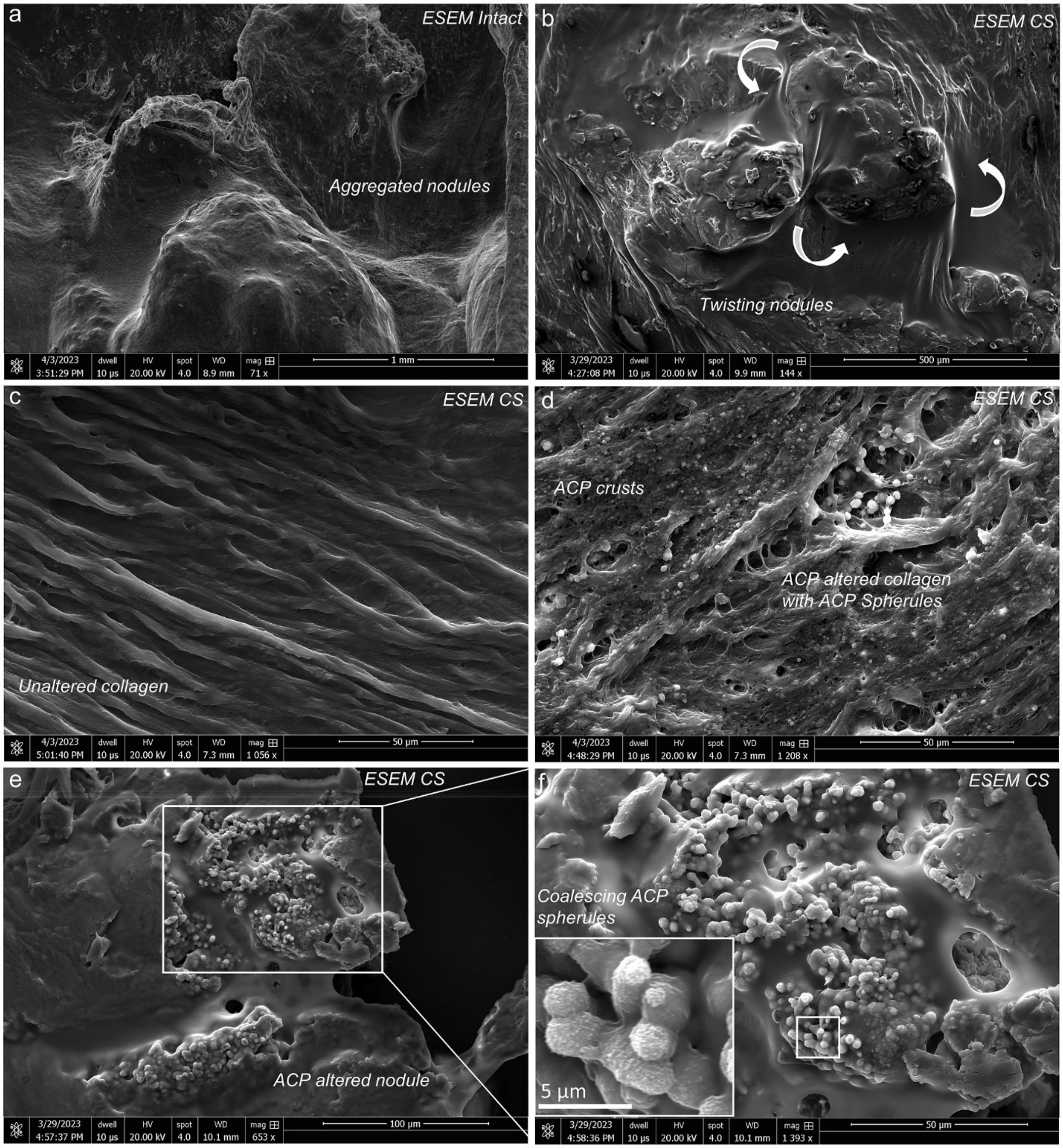

Supplementary Data Fig. 1 | ESEM analyses of ACP calcification of collagen fibers within the fibrosa layer of human aortic valve leaflets. Contextual expansion and additional images to complement Figure 5. **a** and **b**, Multiple nodules contained by collagen on the aortic side of the fibrosa layer. White arrows illustrate nodule twisting and turning during leaflet flexure. **c**, Original unaltered collagen fibers (expanded view of Fig. 5). **d**, Calcified collagen exhibiting individual and coalescing ACP spherules and crusts. **e** and **f**, ACP spherules comprising core of nodules. ESEM = environmental scanning electron microscope. ACP = amorphous calcium phosphate.

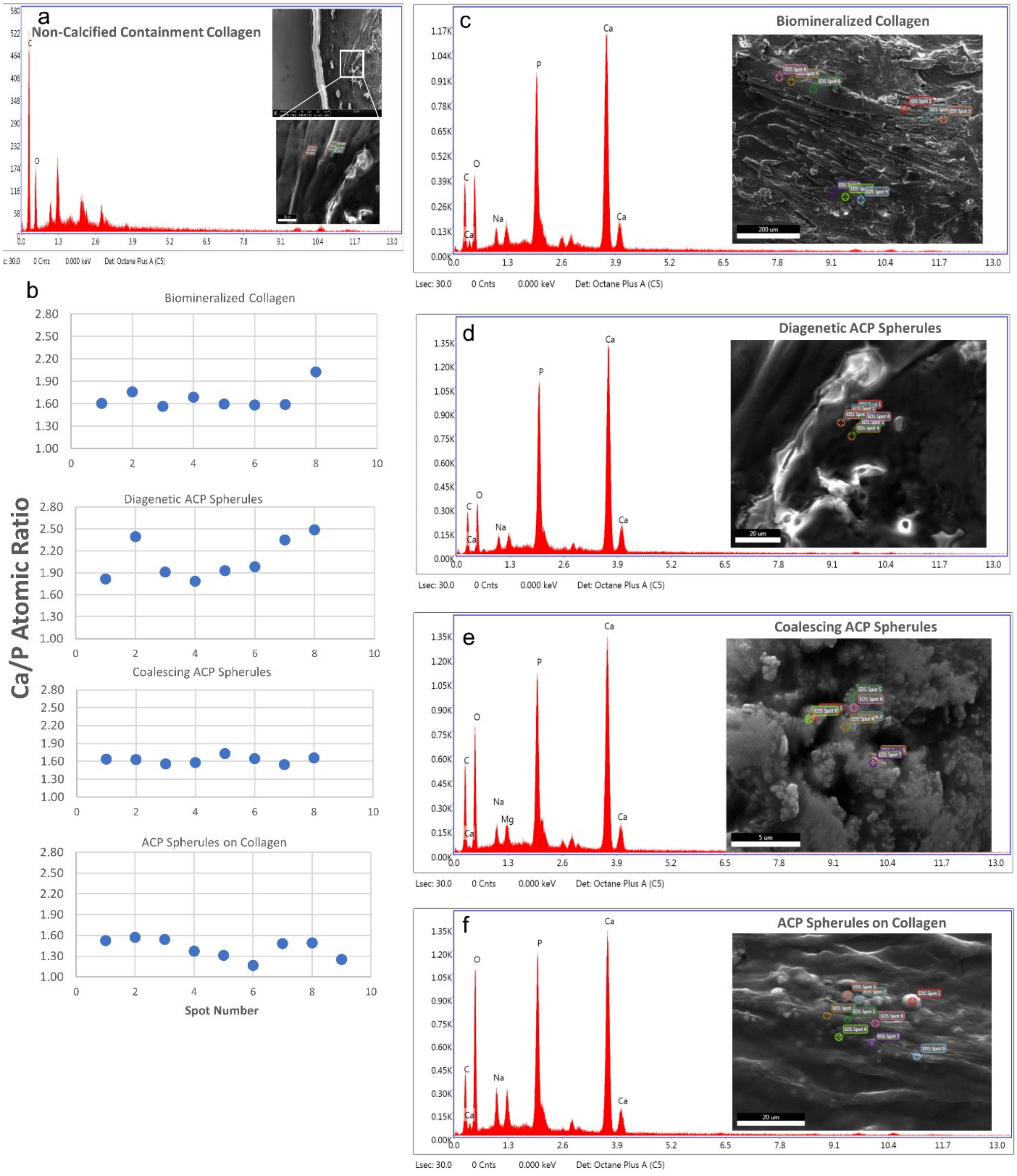

Supplementary Data Fig. 2 | Representative ESEM-EDAX analysis of calcium phosphates in human aortic valve leaflets. a-h, Representative EDAX spot analyses (n = 9) showing spectral peaks for calcium (Ca), phosphate (P) and magnesium (Mg). **b**, Ca/P atomic ratios calculated from the analyzed EDAX spot spectra. Specific locations for each spot analysis are presented as a title in each figure. ESEM = environmental scanning electron microscope. EDAX = energy dispersive X-ray spectroscopy. ACP = amorphous calcium phosphate.

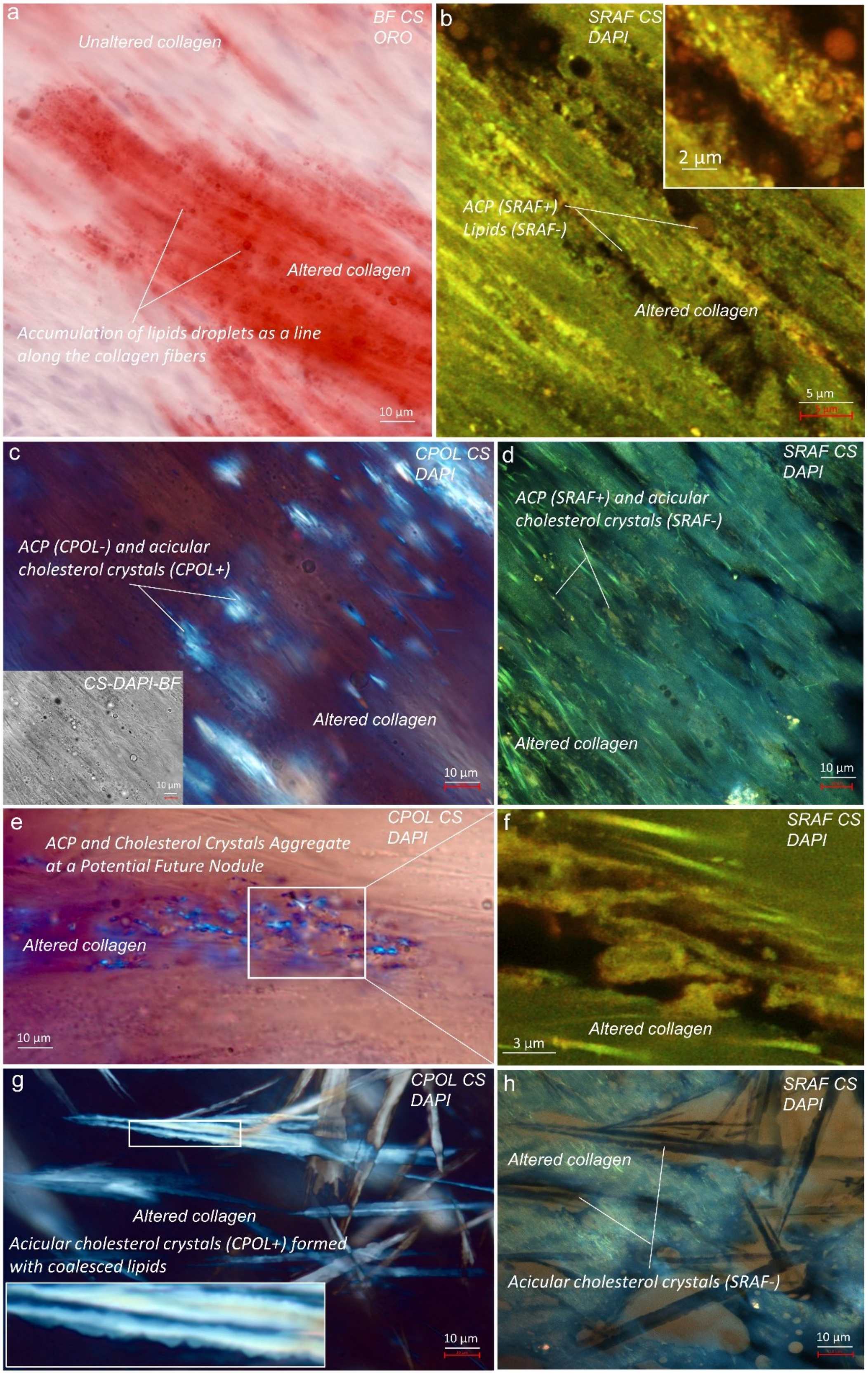

a
BF CS
ORO
Unaltered collagen
Altered collagen
Accumulation of lipids droplets as a line along the collagen fibers
10 µm
b
SRAF CS
DAPI
2 µm
ACP (SRAF+)
Lipids (SRAF-)
Altered collagen
5 µm
c
CPOL CS
DAPI
ACP (CPOL-) and acicular cholesterol crystals (CPOL+)
CS-DAPI-BF
Altered collagen
10 µm
d
SRAF CS
DAPI
ACP (SRAF+) and acicular cholesterol crystals (SRAF-)
Altered collagen
10 µm
e
CPOL CS
DAPI
ACP and Cholesterol Crystals Aggregate at a Potential Future Nodule
Altered collagen
10 µm
f
SRAF CS
DAPI
Altered collagen
3 µm
g
CPOL CS
DAPI
Altered collagen
Acicular cholesterol crystals (CPOL+) formed with coalesced lipids
10 µm
h
SRAF CS
DAPI
Altered collagen
Acicular cholesterol crystals (SRAF-)
10 µm

Supplementary Data Fig. 3 | ACP and cholesterol calcification of collagen fibers within the fibrosa layer of human aortic valve leaflets. Contextual and expanded views for the data images presented in main Figure 5. **a**, Lipid droplets aligned along collagen fibers (dark red areas) adjacent to unaltered collagen layers (pink areas). **b**, ACP and lipid droplets aligned along a calcified collagen fiber similar to the area in **a**. **c** and **d**, Paired images of ACP and cholesterol showing differential response in CPOL and SRAF. **e** and **f**, Paired CPOL and SRAF images showing a region of ACP and cholesterol crystal aggregation in early stages of nodule formation. **g** and **h**, Paired CPOL and SRAF images showing clusters of acicular cholesterol crystals. ACP = amorphous calcium phosphate. CPOL = circular polarization. CS = cryosection. BF = bright field. SRAF = super resolution autofluorescence. DAPI = 4′,6-diamidino-2-phenylindole stain for DNA.

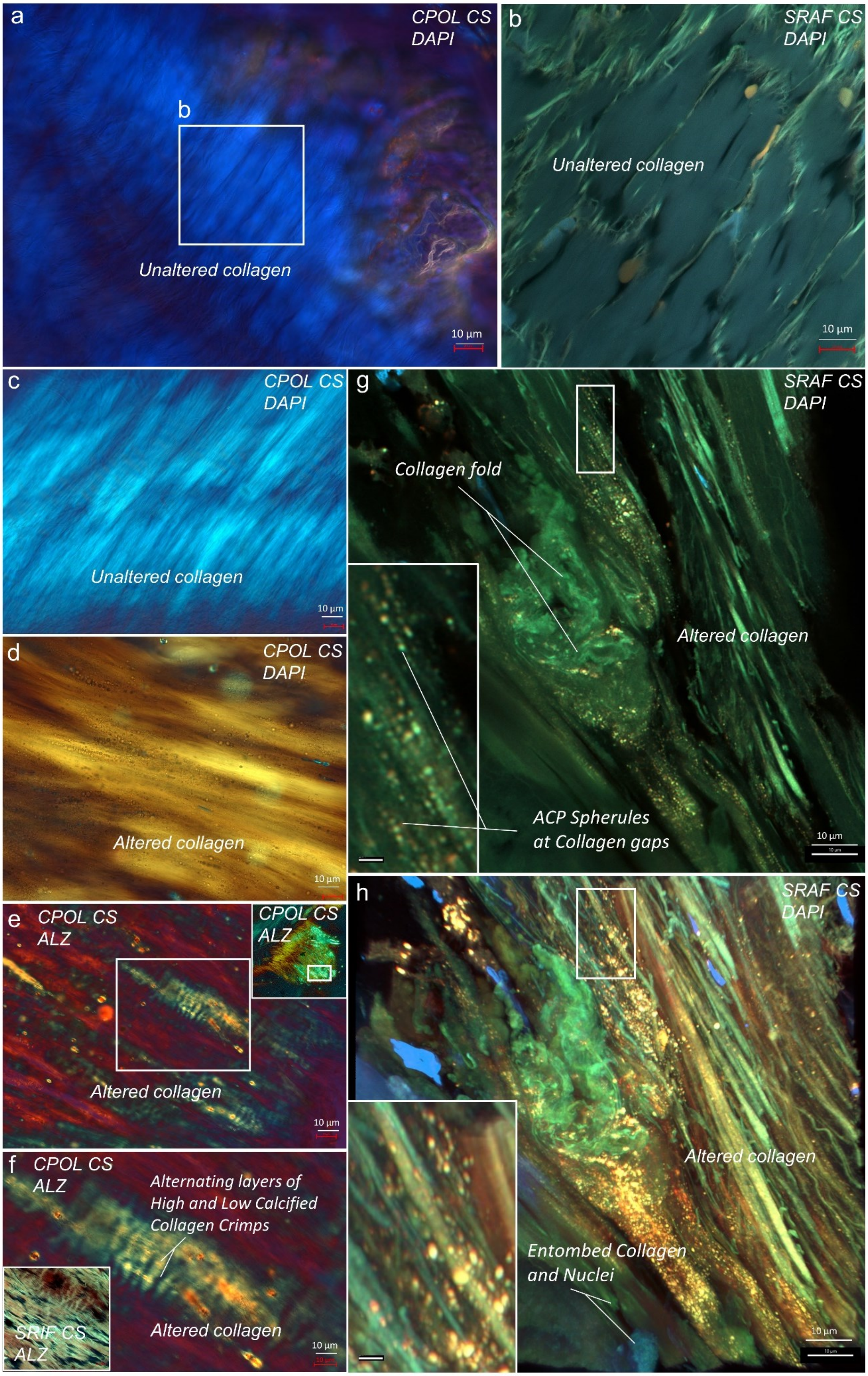

a
CPOL CS
DAPI
b
Unaltered collagen
10 µm
b
SRAF CS
DAPI
Unaltered collagen
10 µm
c
CPOL CS
DAPI
Unaltered collagen
10 µm
d
CPOL CS
DAPI
Altered collagen
10 µm
e
CPOL CS
ALZ
CPOL CS
ALZ
Altered collagen
10 µm
f
CPOL CS
ALZ
Alternating layers of
High and Low Calcified
Collagen Crimps
SRIF CS
ALZ
Altered collagen
10 µm
g
SRAF CS
DAPI
Collagen fold
Altered collagen
ACP Spherules
at Collagen gaps
10 µm
h
SRAF CS
DAPI
Altered collagen
Entombed Collagen
and Nuclei
10 µm

Supplementary Data Fig. 4 | ACP calcification of collagen fibers within the fibrosa layer of human aortic valve leaflets. Contextual and complementary modality images for main Figures 5 and 6. **a** and **b**, Paired CPOL and SRAF images of unaltered collagen fibers (expanded views for Figs. 5c and d). **c**, CPOL image of unaltered and mimetically altered collage fibers in **d**. **e** and **f**, Contextual and expanded views of CPOL images for Figure 2i. Note the alternating low and high calcification pattern reflecting folding along the crimped collagen fibers (yellow to orange ACP; magenta to green ACP). **g** and **h**, expanded view of an SRAF optical section (single slice in **g**) and 3D projection of ACP spherules in altered collagen fibers from a zoomed-in region in Figure 6f showing the nodule-collagen interface. Note the presence of collagen folds, as well as distinct spacing of ACP spherules along the collagen fibrils along with the entombed collagen and nuclei in the developing nodule. ACP = amorphous calcium phosphate. CPOL = circular polarization. SRAF = super resolution autofluorescence. 3D = three-dimensional. CS = histology cryosection. BF = bright field. ESEM = environmental scanning electron microscope. SRAF = super resolution autofluorescence. SRIF = super resolution induced fluorescence. ALZ = Alizarin Red S stain for calcium. DAPI = 4′,6-diamidino-2-phenylindole stain for DNA.

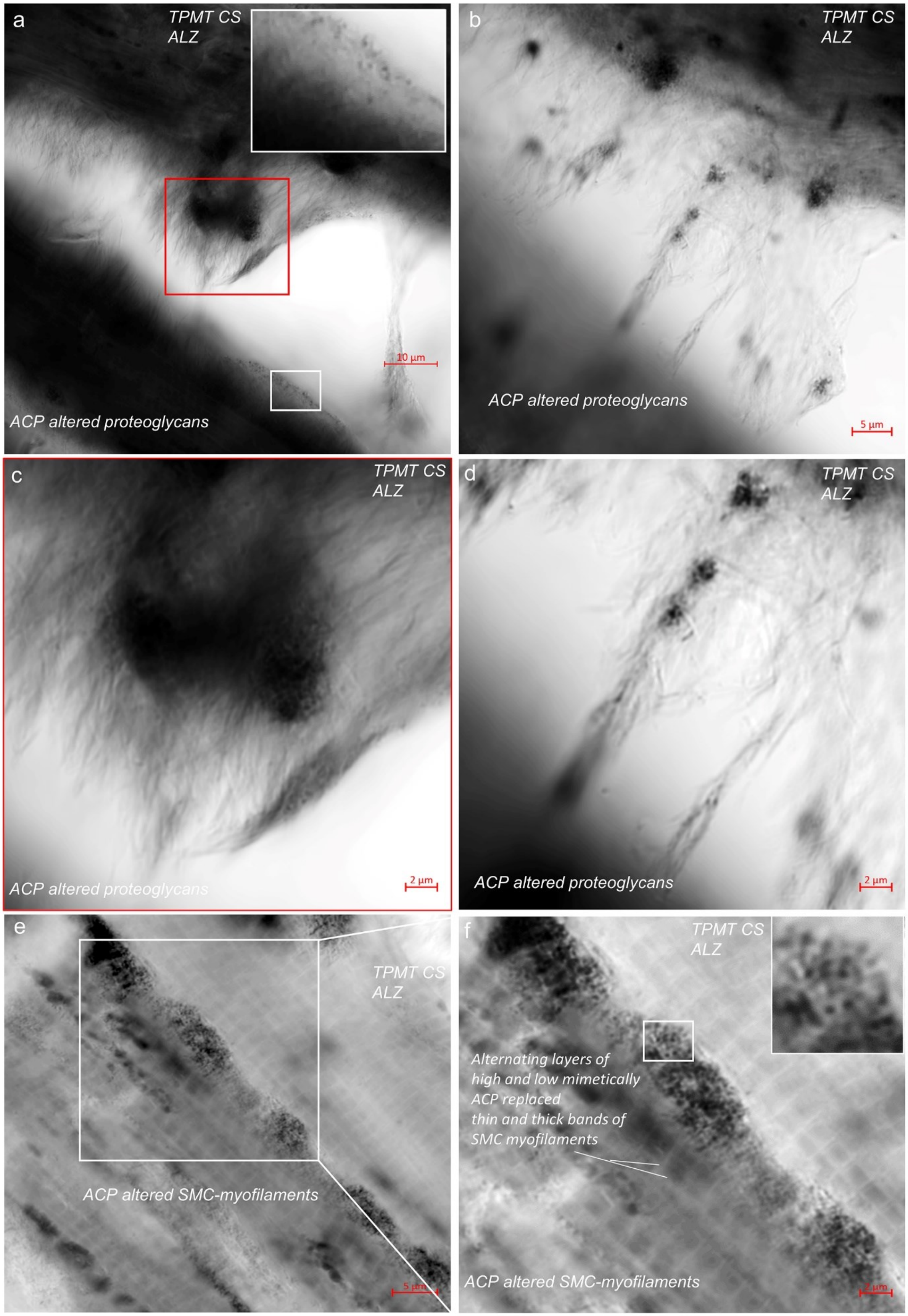
a
TPMT CS
ALZ
10 µm
ACP altered proteoglycans
b
TPMT CS
ALZ
ACP altered proteoglycans
5 µm
c
TPMT CS
ALZ
ACP altered proteoglycans
2 µm
d
TPMT CS
ALZ
ACP altered proteoglycans
2 µm
e
TPMT CS
ALZ
ACP altered SMC-myofilaments
5 µm
f
TPMT CS
ALZ
Alternating layers of
high and low mimetically
ACP replaced
thin and thick bands of
SMC myofilaments
ACP altered SMC-myofilaments
2 µm

Supplementary Data Fig. 5 | TPMT imaging of ACP calcification of smooth muscle cell myofilaments and associated proteoglycans within the fibrosa layer of human aortic valve leaflets. Contextual and expanded views for the data images presented in Figures 5k and l. **a-d**, ACP spherules calcifying proteoglycan strands showing individual, coalesced and aggregated spherules. **e** and **f**, Differential ACP calcification within thin (actin) and thick (myosin) banding of SMC myofilaments. Note higher (darker) extent of mimetic replacement within thick bands compared to thin (lighter) bands together. In addition, ACP spherule clusters align (white boxes) along SMC myofilaments. ACP = amorphous calcium phosphate. SMC = smooth muscle cells. TPMT = transmitted light photomultiplier tube. ALZ = Alizarin Red S stain for calcium.

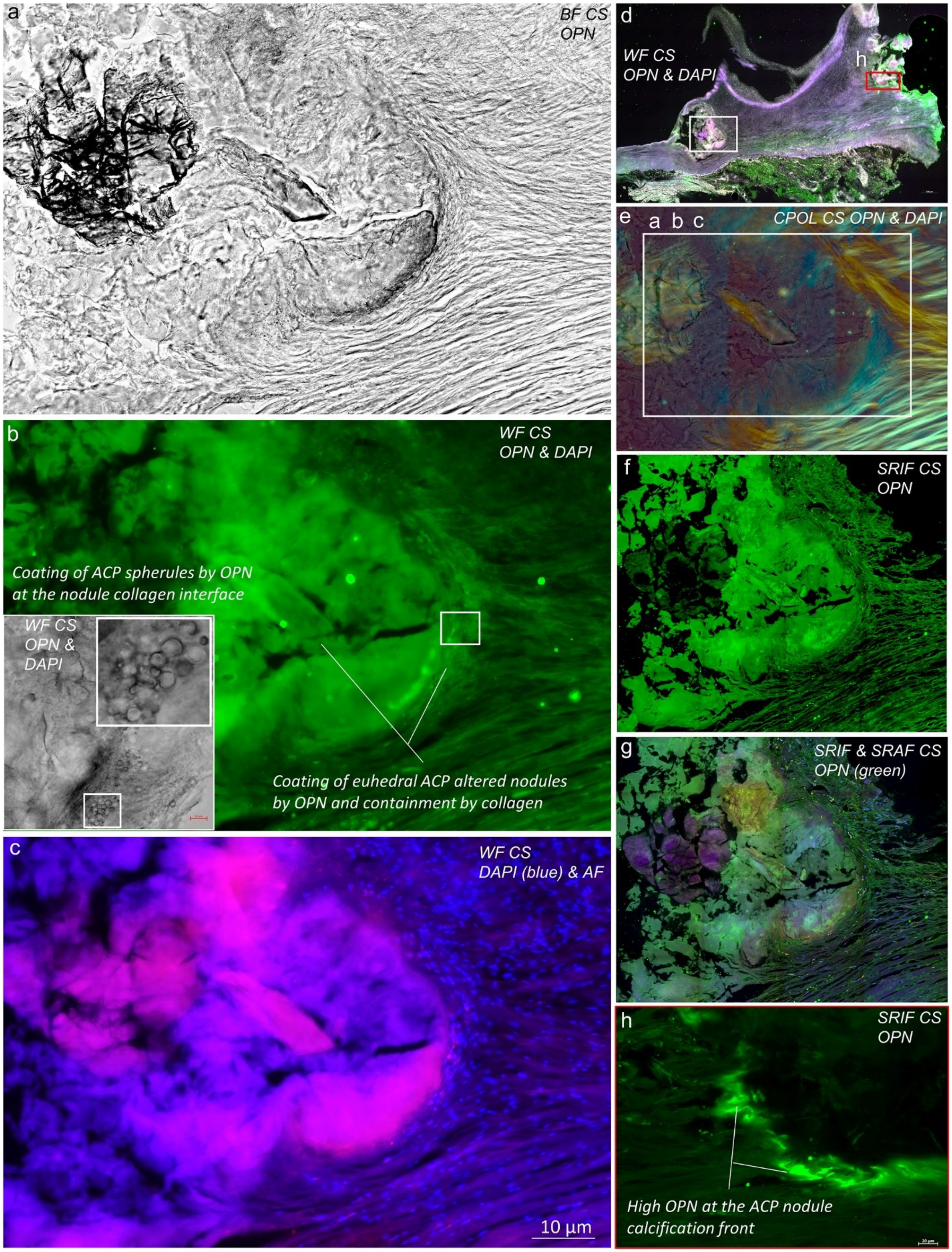

a
BF CS
OPN
b
WF CS
OPN & DAPI
Coating of ACP spherules by OPN
at the nodule collagen interface
WF CS
OPN &
DAPI
Coating of euhedral ACP altered nodules
by OPN and containment by collagen
c
WF CS
DAPI (blue) & AF
10 µm
d
WF CS
OPN & DAPI
h
e
a b c
CPOL CS OPN & DAPI
f
SRIF CS
OPN
g
SRIF & SRAF CS
OPN (green)
h
SRIF CS
OPN
High OPN at the ACP nodule
calcification front

Supplementary Data Fig. 6 | Osteopontin (OPN) protein concentrations and distributions at the collagen-nodule interface within the fibrosa layer of human aortic valve leaflets. Contextual and expanded views of the images presented in Figures 6d. **a-c**, Paired images showing the distribution of OPN within the ACP nodule-collagen interface. High magnification in b inset showing ACP spherules also coated with OPN. **d**, Contextual image for all other high magnifications in Figure 8. **e-g**, Contextual expansions of paired CPOL, OPN and AF images showing the extent of OPN localization at the nodule-collagen interface. **h**, Nodule exhibiting high concentrations of OPN where ACP calcification is occurring at the nodule-collagen interface. ACP = amorphous calcium phosphate. CPOL = circular polarization. SRIF = super resolution induced fluorescence. CS = histology cryosection. BF = bright field. WF = widefield. AF = autofluorescence. WF = wide field. SRIF = super resolution induced fluorescence. OPN = Alexa 647 antibody stain for osteopontin. DAPI = 4′,6-diamidino-2-phenylindole stain for DNA.

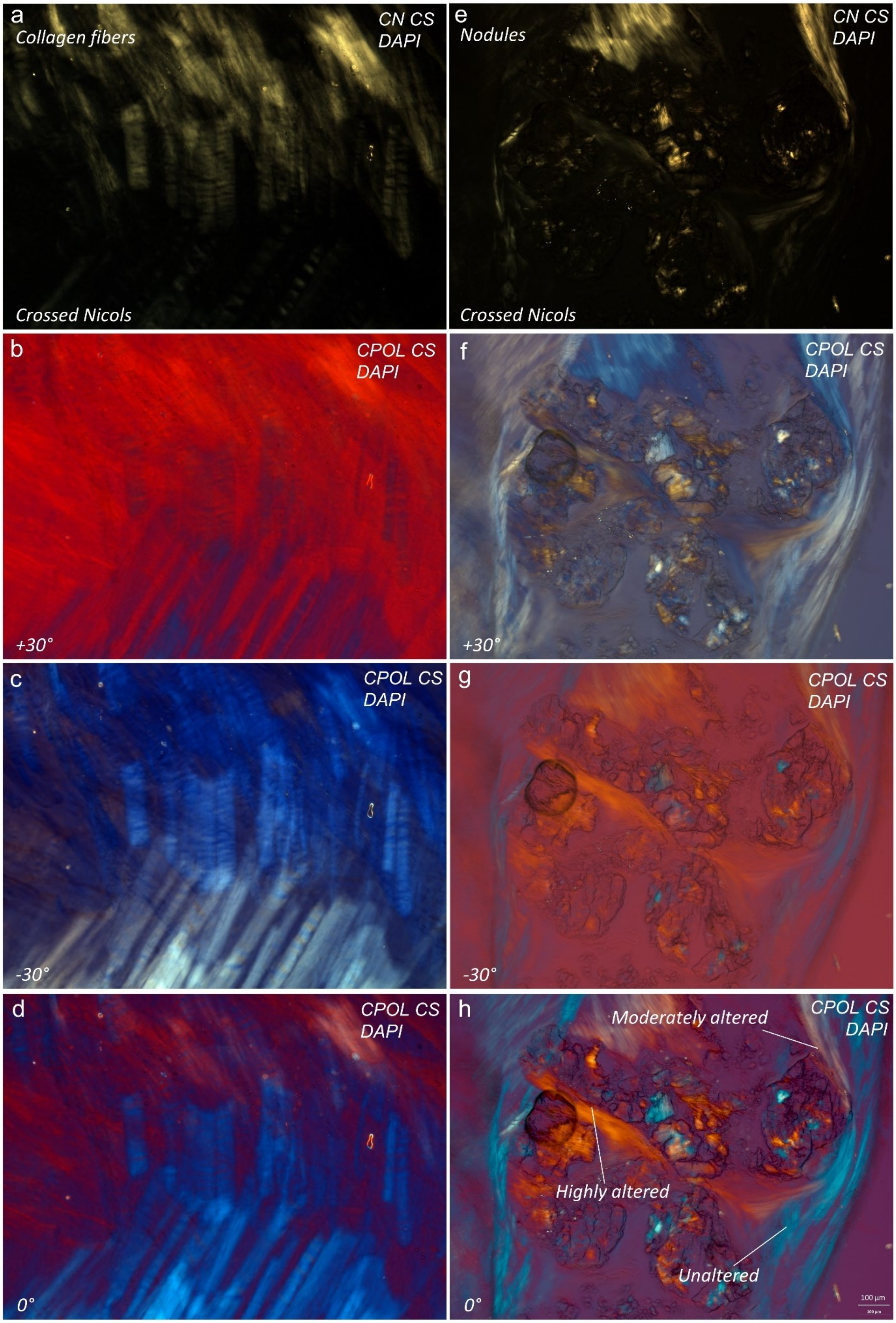

a
Collagen fibers
CN CS
DAPI
Crossed Nicols
e
Nodules
CN CS
DAPI
Crossed Nicols
b
CPOL CS
DAPI
+30°
f
CPOL CS
DAPI
+30°
c
CPOL CS
DAPI
-30°
g
CPOL CS
DAPI
-30°
d
CPOL CS
DAPI
0°
h
Moderately altered
CPOL CS
DAPI
Highly altered
Unaltered
0°
100 µm

Supplementary Data Fig. 7 | Calibration and comparison of crossed Nicol (POL) and circular polarization (CPOL) imaging modalities used in interpreting the extent of calcification based on the Zeiss Michael-Levy birefringence chart in human aortic valve leaflets. a-d, The crossed Nicole modality could not reveal all collagen fibers because the technique is dependent on the orientation of molecules with respect to collagen fiber axial polarization angle (**a**). A CPOL polarizer set at 0° separated blue unaltered collagen fibers from altered collagen fibers higher order birefringence. **e-h**, In the case of nodules, the crystalline cholesterol crystals and thick calcified collagen fibers are detected under crossed nicols. This technique clearly identifies unaltered (blue birefringence), moderately altered (yellow-orange birefringence) and highly altered (bright orange birefringence) collagen fibers, as well as highly birefringent cholesterol crystals (**e**; again, only with the 0°). As a result, CPOL birefringence at 0° is consistently used throughout the manuscript following color terminology presented in Zeiss Michael-Levy birefringence chart. CS = histology cryosection. CPOL = circular polarization. POL = crossed Nicol polarization.

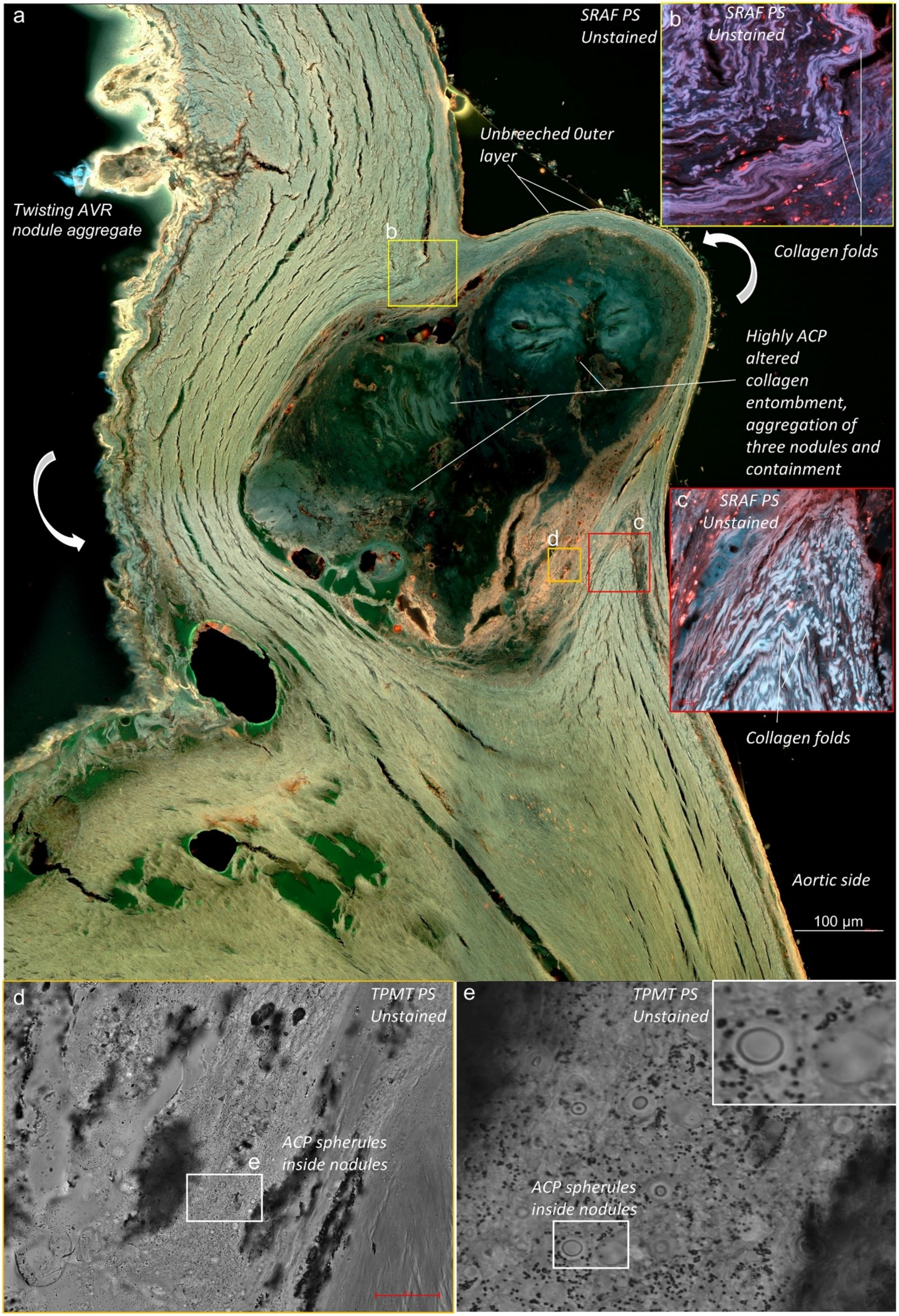
a
SRAF PS
Unstained
b
SRAF PS
Unstained
Unbreeched 0uter
layer
Twisting AVR
nodule aggregate
b
Collagen folds
Highly ACP
altered
collagen
entombment,
aggregation of
three nodules and
containment
c
SRAF PS
Unstained
d
c
Collagen folds
Aortic side
100 µm
d
TPMT PS
Unstained
ACP spherules
inside nodules
e
e
TPMT PS
Unstained
ACP spherules
inside nodules

Supplementary Data Fig. 8 | Nodule twisting, collagen folding and nodule containment in unstained petrographic thin sections of human aortic valve leaflets. Contextual and expanded high magnification views for images presented in Figures 7a and b. **a**, Contextual view of highly calcified collagen entombment, aggregation of multiple nodules and its containment. Nodule twisting motions (white arrows) are recorded by folded collagen fibers on each side of the nodule (colored boxes). **d** and **e**, Entombed ACP spherules occur inside the early development stages of a nodule, with the ACP spherule exhibiting characteristic concentric zonation. ACP = amorphous calcium phosphate. PS = epoxy impregnated petrographic section. SRAF = super resolution autofluorescence. TPMT = transmitted light photomultiplier tube.

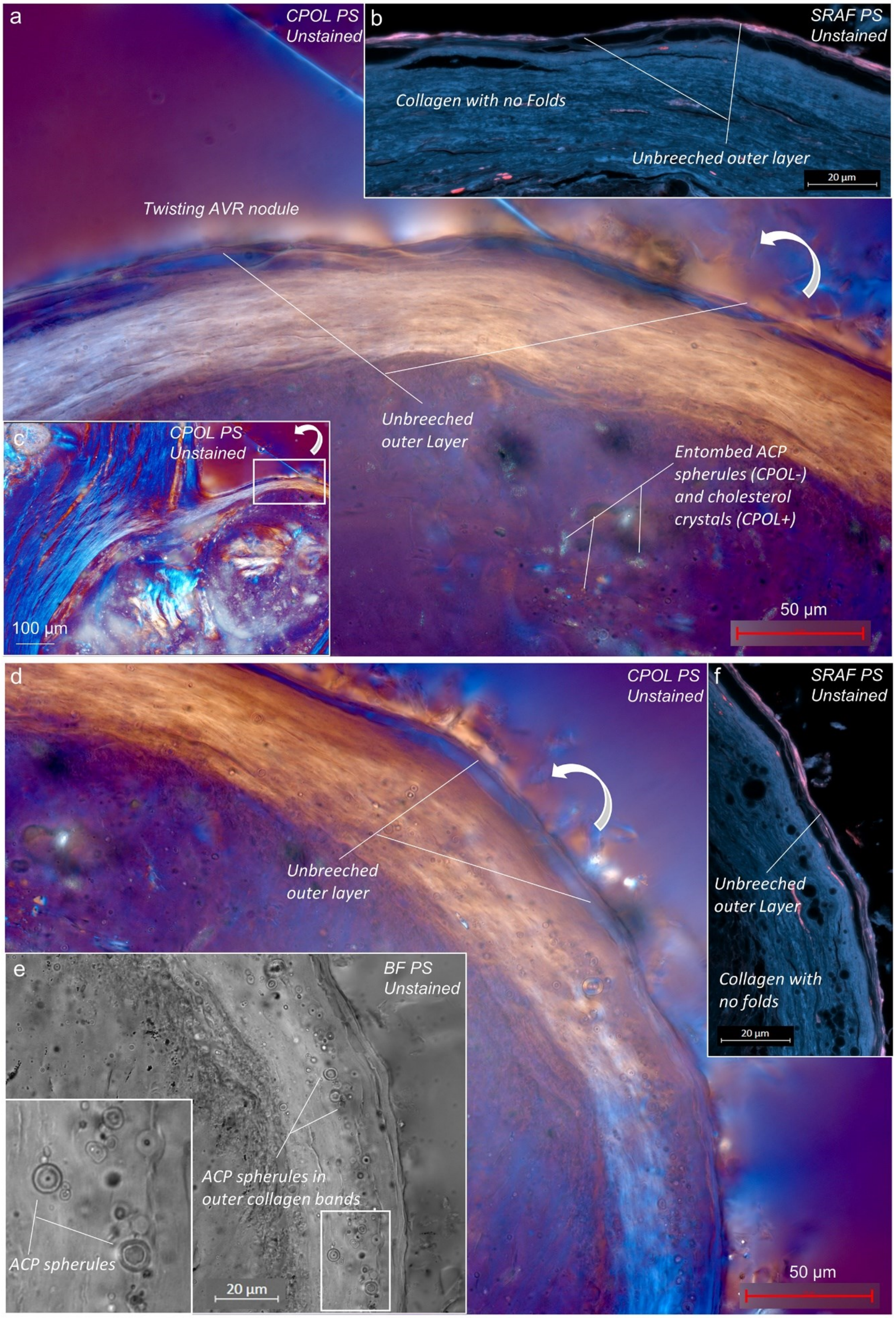
a
CPOL PS
Unstained
b
SRAF PS
Unstained
Collagen with no Folds
Unbreeched outer layer
20 µm
Twisting AVR nodule
Unbreeched
outer Layer
c
CPOL PS
Unstained
100 µm
Entombed ACP
spherules (CPOL-)
and cholesterol
crystals (CPOL+)
50 µm
d
CPOL PS
Unstained
f
SRAF PS
Unstained
Unbreeched
outer layer
Unbreeched
outer Layer
Collagen with
no folds
20 µm
e
BF PS
Unstained
ACP spherules in
outer collagen bands
ACP spherules
20 µm
50 µm

Supplementary Data Fig. 9 | Containment of ACP nodules within collagen fibers in an unstained petrographic thin section of human aortic valve leaflets. Contextual and expanded high magnification views for the images presented in Figures 7a and b and Supplementary Data Figure 8. **a-c**, Outermost edge of an ACP nodule on the aortic side of the fibrosa layer contained by unbreeched layers unaltered collagen fibers. Collagen fibers immediately adjacent to the nodule contain ACP spherules (**e**). Cholesterol crystals are entombed within the developing nodule. Images **a** and **d** are adjacent to the growing margin of the aggregated nodule. Note the SRAF images in the inset in **b** and **f**, show no collagen folds at regions exposed to aortic side. The folds are seen clearly at the nodule-leaflet interface as in Fig 5. a, b. ACP = amorphous calcium phosphate. PS = epoxy impregnated petrographic section. CPOL = circular polarization. SRAF = super resolution autofluorescence. BF = bright field.

Supplementary Data Fig. 10 | Contextual expanded views for Fig. 2 showing Raman spectroscopy results of nodules within human aortic valve leaflet tissues in histology cryosections embedded in ultrapure water. The nine locations of the spectra were taken randomly using the WiTec Alpha Raman system (more details are in the main manuscript methods). The contextual locations shown in a paired BF (black and white) and CPOL (color) tiled images for the spectra presented in (Fig. 2). BF = brightfield. CPOL = circular polarization.

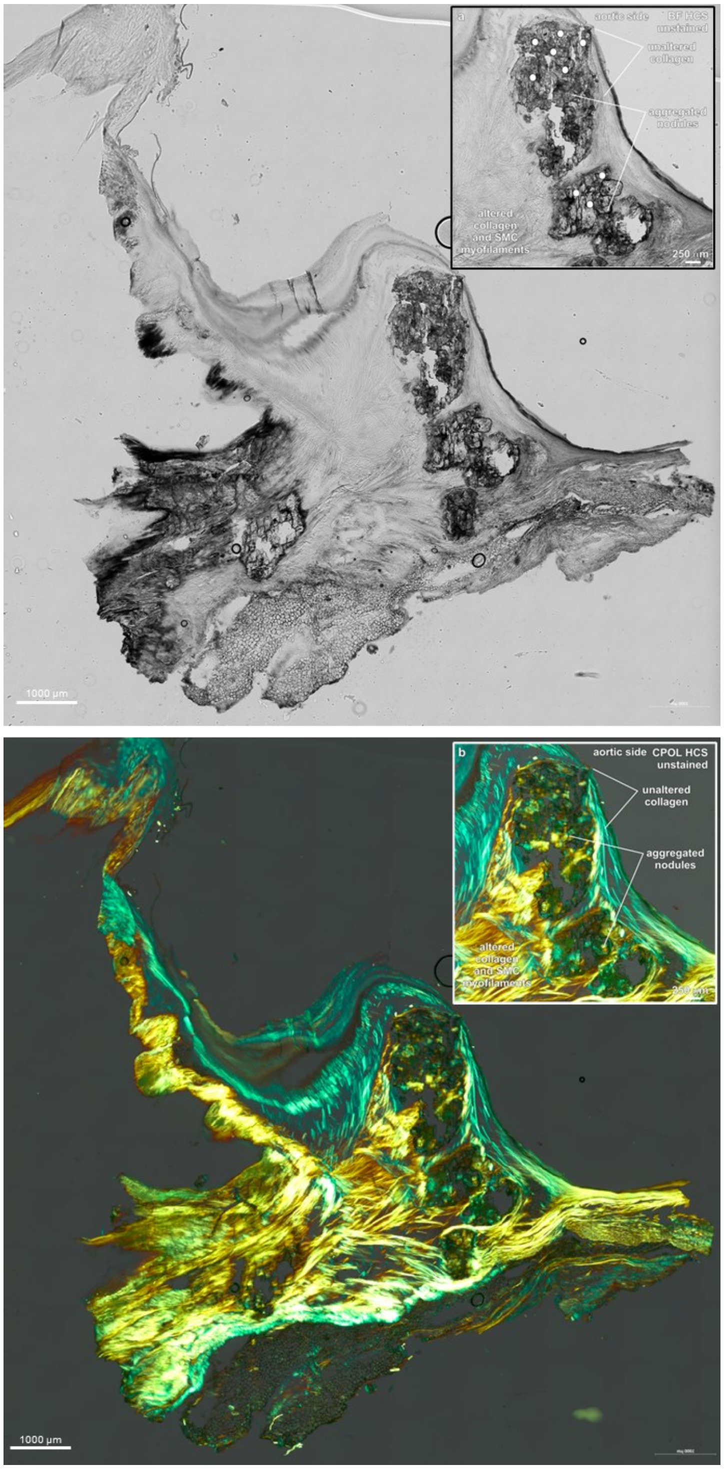

Supplementary Movies Guide

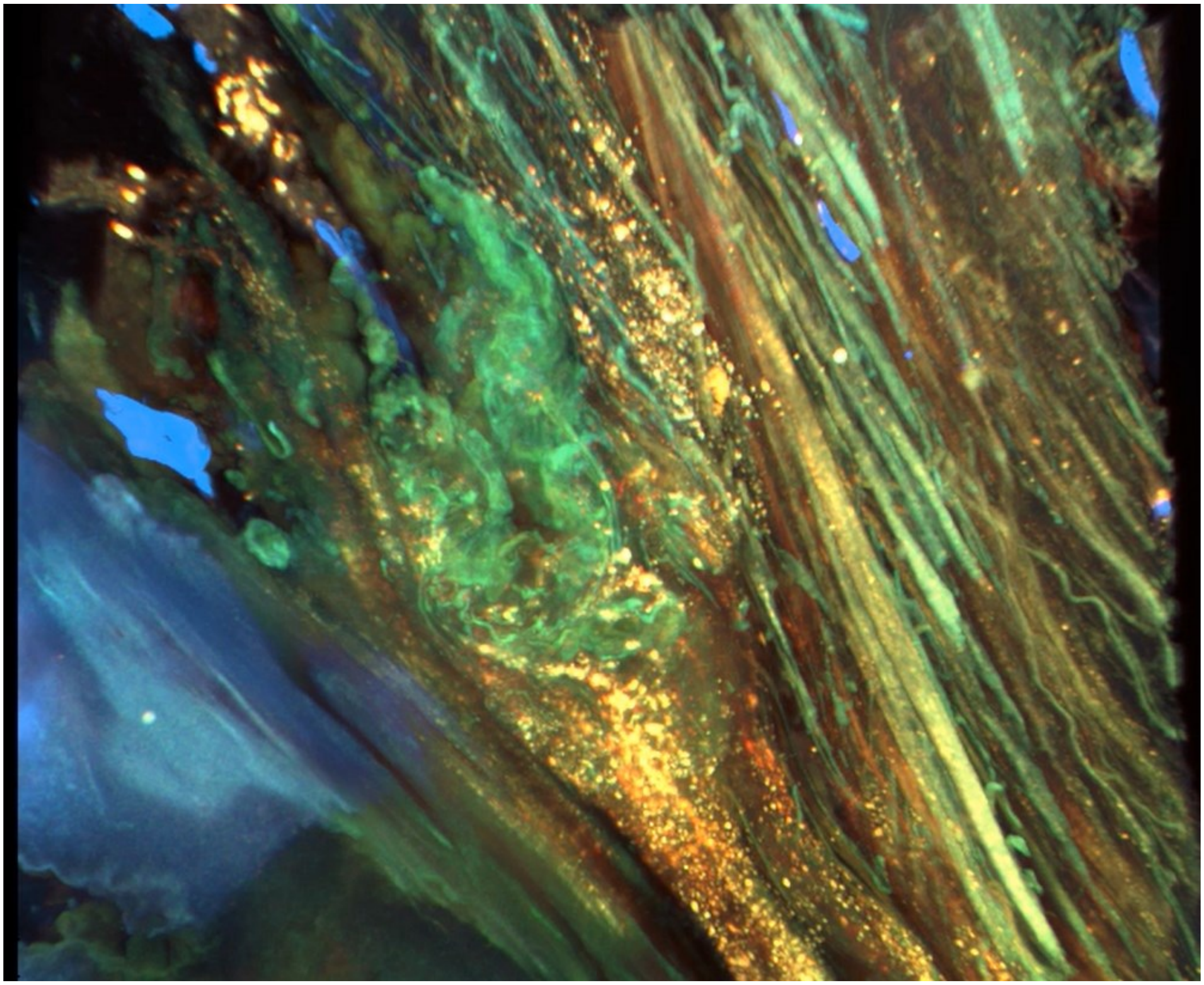

Supplementary Movie 1. Layers of crimped and folded collagen fibers (upper right) being diagenetically incorporated into an amorphous calcium phosphate (ACP) nodule (lower left) within the fibrosa layer of a human aortic valve leaflet. Video depicts the three-dimensional (3D) structure of the contact between an ACP nodule and altered collagen fibers as revealed by 3-channel (red, green, blue; RGB) super resolution autofluorescence (SRAF) images taken through a series of optical sections taken vertically through the Z direction of the histology cryosection. From the same region shown in Figure 6i and Supplementary Data Figures 4g and h.

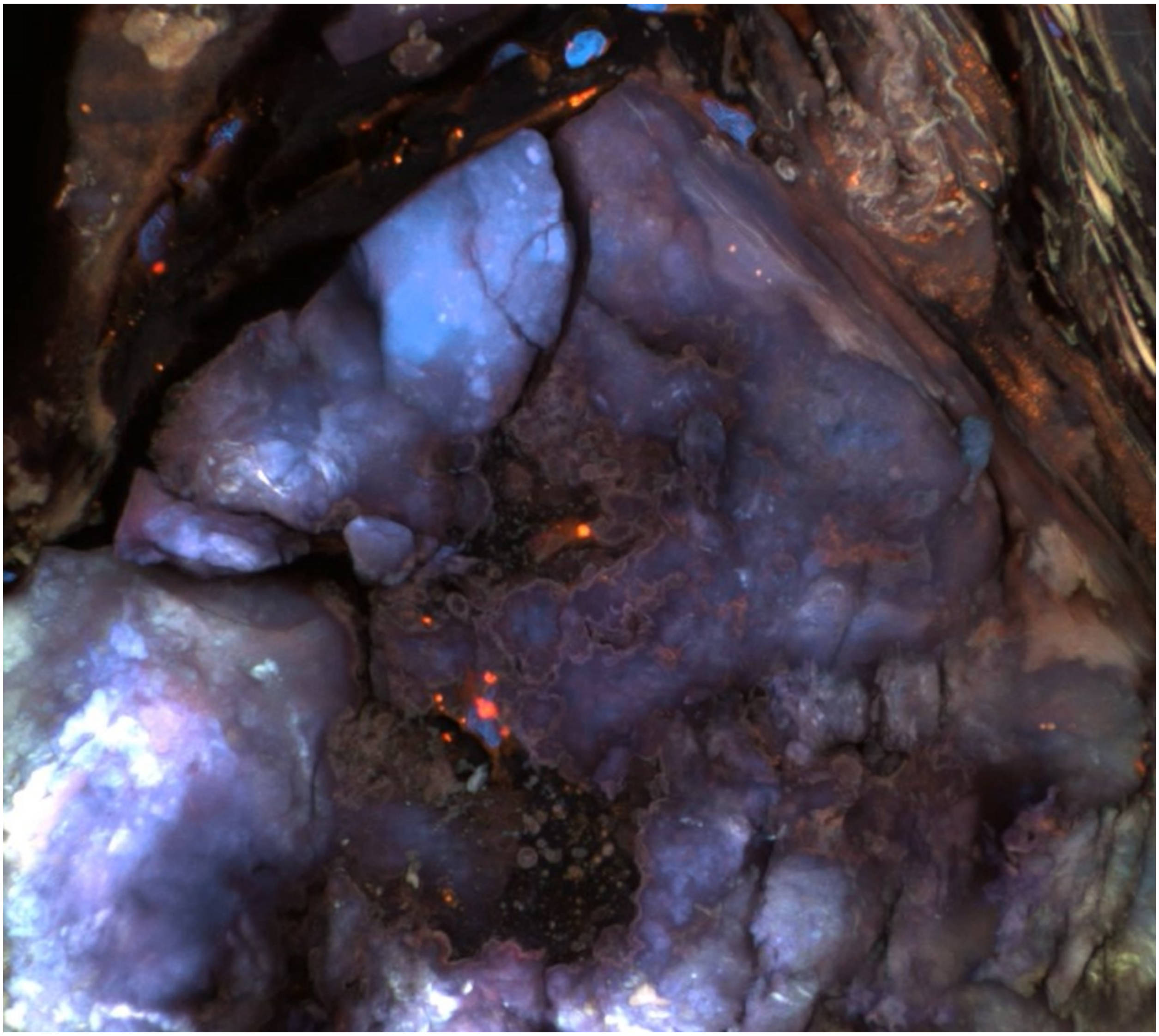

Supplementary Movie 2. Lower magnification of Video 1 cryosection region (upper right), showing layers of amorphous calcium phosphate (ACP) spherules (light beige fluorescence) that coalesce to form ACP nodules (dark to light lavender fluorescence) within the fibrosa layer of a human aortic valve leaflet. Video depicts the three-dimensional (3D) structure of an ACP nodule as revealed by 3-channel (red, green, blue; RGB) super resolution autofluorescence (SRAF) images taken through a series of optical sections taken vertically through the Z direction of the histology cryosection. Coalescing ACP spherules begin to attain atomic order and form early euhedral shapes at the top of the ACP nodule. From the same cryosection region shown in Figure 6i and Supplementary Data Figures 4g and h.

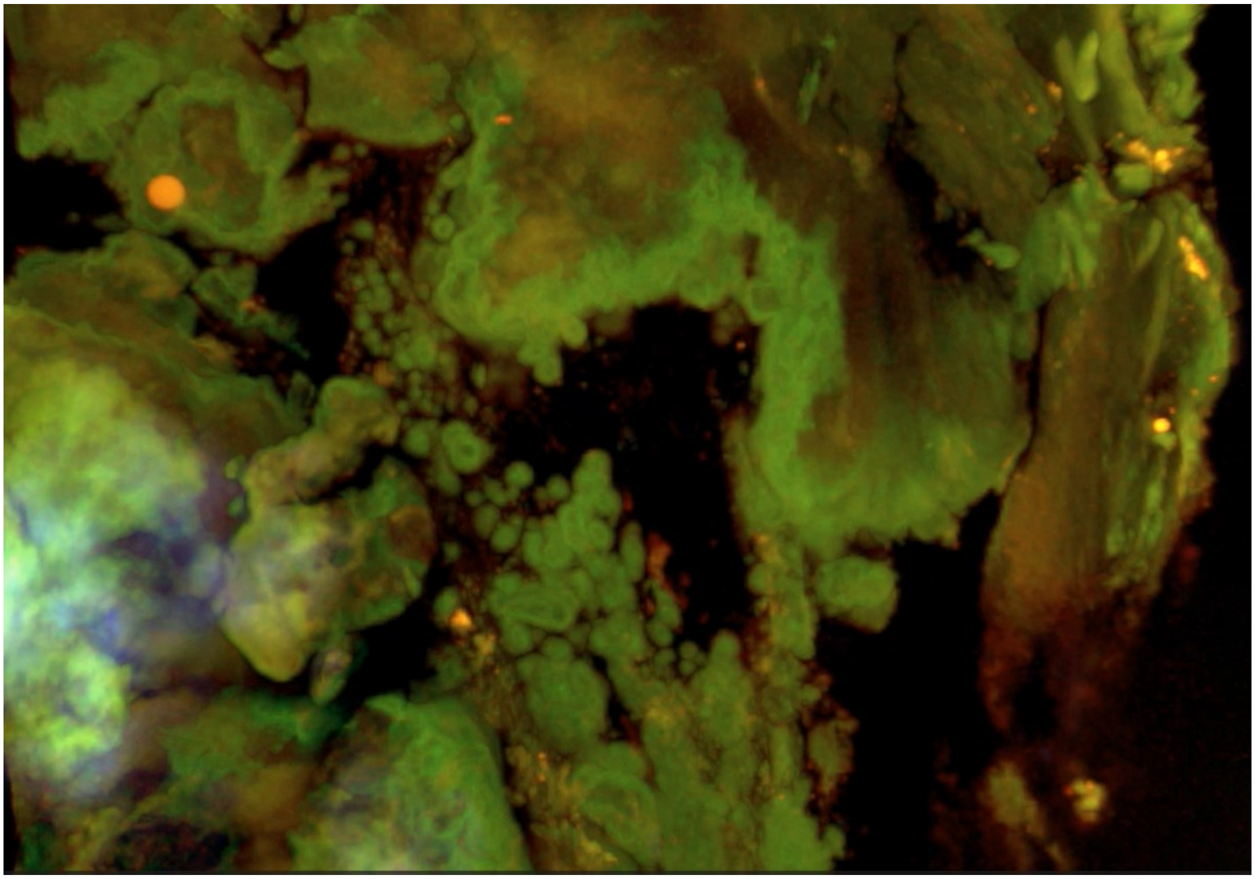

Supplementary Movie 3. Higher magnification of the central portion of the amorphous calcium phosphate (ACP) nodule in Video 2 showing layers of spherules (light green to beige fluorescence) that coalesce to form ACP nodules within the fibrosa layer of a human aortic valve leaflet. Video depicts the three-dimensional (3D) structure of an ACP nodule as revealed by 3-channel (red, green, blue; RGB) super resolution autofluorescence (SRAF) images taken through a series of optical sections taken vertically through the Z direction of the histology cryosection. From the same cryosection region shown in Figure 6e, f, and i.
